# Careful Examination of a Novel Azobenzene Paroxetine Derivative and Its Interactions With Biogenic Amine Transporters

**DOI:** 10.1111/jnc.70068

**Published:** 2025-04-24

**Authors:** Dominik Dreier, Oliver John V. Belleza, Katharina Schlögl, Stefanie Kickinger, Eva Hellsberg, Felix P. Mayer, Walter Sandtner, Philipp Mikšovsky, Matthias Schittmayer, Yuntao Hu, Kathrin Jäntsch, Marion Holy, Gerhard F. Ecker, Harald H. Sitte, Marko D. Mihovilovic

**Affiliations:** ^1^ Institute of Applied Synthetic Chemistry TU Wien Vienna Austria; ^2^ Institute of Pharmacology, Center for Physiology and Pharmacology Medical University of Vienna Vienna Austria; ^3^ Department of Pharmaceutical Chemistry University of Vienna Vienna Austria; ^4^ Institute for Chemical Technologies and Analytics TU Wien Vienna Austria; ^5^ Hourani Center for Applied Scientific Research Al‐Ahliyya Amman University Amman Jordan; ^6^ Center for Addiction Research and Science Medical University Vienna Vienna Austria

**Keywords:** azobenzene, GABA, neurotransmitter transporters, norepinephrine, off‐target effect, serotonin

## Abstract

The serotonin transporter (SERT) belongs to the family of neurotransmitter sodium symporters (NSS), together with other neurotransmitter transporters for norepinephrine, dopamine, glycine, and GABA. The main physiological role of SERT is the retrieval of previously released serotonin from the synaptic cleft. Thereby, SERT plays an important role in regulating the extracellular serotonin concentration and maintaining serotonergic neurotransmission. This process can be influenced by molecules acting as serotonin uptake inhibitors, like paroxetine. Here, we report the development of a novel photoswitchable paroxetine derivative and its pharmacological interaction profile with SERT as a tool compound for the light‐induced control of SERT. Based on the azo‐extension strategy, the photoswitchable moiety was formed at the former position of the fluoro substituent in paroxetine. The resulting azo‐paroxetine (**9**) was easily and reversibly switched between active (*Z*) and inactive (*E*) configurations and remained stable between these configurations: serotonin uptake was inhibited more than 12 times more potently by the active (*Z*)‐configuration having a sub μM IC_50_ value. This was supported by electrophysiological patch‐clamp recordings in the whole‐cell configuration and docking studies. No significant toxic impact of azo‐paroxetine (**9**) and no off‐target activity at the norepinephrine transporter (NET), human GABA transporter subtypes 1 and 3, and rat GAT1 were observed. Our results demonstrate that the activity of SERT can be reversibly manipulated by the optopharmacological agent azo‐paroxetine (**9**). This compound can thus be applied as a tool for the selective manipulation of SERT in central or peripheral investigations, further benefiting from its low probability for compound‐related off‐target effects.

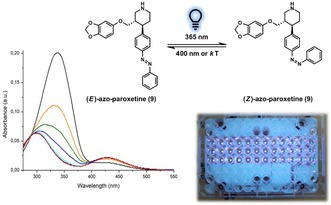

## Graphical abstract

The main physiological role of the serotonin transporter (SERT) is to retrieve previously released serotonin from the synaptic cleft, thus regulating serotonergic signaling. Serotonin uptake inhibitors, like paroxetine, can influence this process. We developed a novel photoswitchable paroxetine derivative, effectively switching between its active (*Z*) and inactive (*E*) configurations. This tool compound enables light‐triggered control of SERT with a significant potency difference between the two isomers and no off‐target activity at several tested related neurotransmitter transporters.

**Abbreviations**
5‐HT: 5‐hydroxytryptamine
DA: dopamine
DAT: dopamine transporter
DCE: dichloroethane
DCM: dichloromethane
DMEM: Dulbecco's modified Eagle's medium
DMF: dimethylformamide
DMSO: dimethylsulfoxide
EAAT: excitatory amino acid glutamate transporter
ee: enantiomeric excess
FCS: fetal calf serum
GABA: gamma‐aminobutyric acid
GAT: gamma‐aminobutyric acid transporter
HEK: human embryonic kidney
HEPES: 4‐(2‐hydroxyethyl)‐1‐piperazineethanesulfonic acid
HRMS: high‐resolution mass spectrometry
IC_50_
: half maximal inhibitory concentration
KHB: Krebs‐HEPES buffer
LED: light‐emitting diode
MM‐GBSA: molecular mechanics generalized born surface area
MPP^+^
: 1‐methyl‐4‐phenylpyridinium ion
MTBE: methyl *tert*‐butyl ether
NET: norepinephrine transporter
NMR: nuclear magnetic resonance
NSS: neurotransmitter sodium symporter
NTT: neurotransmitter transporters
PDB: protein data bank
PSS: photostationary state
RMSD: root mean square deviation
RRID: research resource identifier
SERT: serotonin transporter
SLC6: solute carrier 6
STR: short tandem repeat
THF: tetrahydrofuran
UHPLC‐TOF‐MS: ultra‐high performance liquid chromatography time‐of‐flight mass spectrometry
UV/Vis: ultraviolet/visible
VSGB: variable‐dielectric generalized Born model

## 1 Introduction

Neurotransmitter transporters (NTTs) translocate their cognate substrates across biological membranes (Kristensen et al. 2011). NTTs expressed at the plasma membrane remove their cognate substrates from the extracellular space and thereby regulate their availability at pre‐ and postsynaptic receptors. NTTs serve as molecular targets for a variety of clinically relevant (e.g., antidepressants) and recreationally administered drugs (e.g., cocaine) that interfere with the NTT‐mediated reuptake process (Sitte and Freissmuth 2015). Dysfunction of the serotonergic system has been linked to a multitude of affective disorders, for instance, anxiety and depression, which correlate with a reduction in extracellular serotonin (Kristensen et al. 2011). Consequently, the serotonin transporter (SERT) represents a major target for antidepressants (Kristensen et al. 2011). However, currently available drugs that interact with SERT are associated with off‐target effects like influence on vascular function and structure and reduced clinical efficacy in a subset of patients. Hence, the development of novel bioactive compounds that exhibit highly selective interactions with SERT is of utmost importance. Recently, X‐ray and Cryo‐EM structures of various antidepressants bound to SERT became available and thus enable the structure–activity relationship‐driven development of novel SERT‐ligands (Coleman and Gouaux 2018; Coleman et al. 2016, 2020, 2019).

Over the last decade, photopharmacology has emerged as a novel method in which small molecules are rendered light‐activatable in a reversible manner through the attachment of photochromic moieties, with azobenzene being the most commonly used (Trads et al. 2019). The gained spatio‐temporal precision can be employed to study dynamic processes in selected tissues and allows for reducing off‐target effects, which ultimately improves the pharmacological profile of a given drug (Lerch et al. 2016). More recently, the first switchable photopharmacological compounds have been developed, which target NTTs for amino acids, such as the inhibitory *gamma*‐aminobutyric acid (GAT1) (Lutz et al. 2018; Quandt et al. 2014), the excitatory amino acid glutamate (EAAT1‐3) (Cheng et al. 2017), and most recently, the SERT (Cheng et al. 2020). However, these drugs have only been tested for their activity at the transporter where the eponymous parent compound binds to. Hence, possible interactions with other transporters remain unknown. Furthermore, the reports available to date only investigated transporters of a single species. Consequently, the existence of potential species differences remains elusive.

Here, we report the design and synthesis of a photoswitchable analog of paroxetine, termed azo‐paroxetine. Paroxetine is a high‐affinity SERT inhibitor used as an antidepressant that is highly selective over the dopamine transporter (DAT) and NET (Ki‐values (nmol/l): SERT: 0.34 ± 0.03, DAT: 268 ± 8, NET: 156 ± 29) and has little affinity at muscarinic receptors (Kristensen et al. 2011; Owens et al. 2001). We rationalized that the recently published crystal structure of human SERT in complex with paroxetine (Coleman and Gouaux 2018; Coleman et al. 2016) serves as an excellent starting scaffold for the design of a photoswitchable SERT ligand. Paroxetine binds to the orthosteric binding site in SERT (Sorensen et al. 2012) with its fluorophenyl moiety located in the subsite C with access to the extracellular vestibule. Hence, this conformational arrangement should allow for (i) the introduction of a photoswitchable moiety onto the paroxetine scaffold and (ii) enabling photopharmacological (*E*)‐to‐(*Z*)‐isomerization, which is expected to result in affinity changes without inducing massive steric clashes. Furthermore, we thoroughly examined the potential cross‐interaction of azo‐paroxetine and azobenzene with different transporters of the solute carrier 6 (SLC6) family to evaluate whether the addition of the azobenzene moiety changes the pharmacological properties of the parental compound. Finally, we investigated the inhibitory potency of the selective SERT inhibitor paroxetine and its photochromic derivative at other biogenic amine transporters as well as GABA transporters and compared different mammalian species.

## 2 Materials and Methods

### 2.1 NMR Annotation of (*E*)‐azo‐Paroxetine (9)

All compounds have been extensively characterized via *R*_f_‐value, HRMS, specific rotation, and enantiomeric excess where applicable, and NMR (see Supporting Information). For the (*E*)‐state of the final compound (**9**), the assignment of protons and carbon atoms in the NMR code was carried out as follows:

^*1*^ *H‐NMR* (*400 MHz, CDCl*_*3*_) *δ* = 1.78–1.93 (m, 2H, H5), 2.16–2.27 (m, 1H, H3), 2.68–2.84 (m, 3H, H2 & H4 & H6), 2.98 (bs, 1H, NH), 3.22–3.28 (m, 1H, H6), 3.43–3.54 (m, 2H, H2 & ‐O‐CH_2_‐), 3.62 (dd, *J* = 9.5, 3.0 Hz, 1H, ‐O‐CH_2_‐), 5.86 (s, 2H, H2″), 6.13 (dd, *J* = 8.5, 2.5 Hz, 1H, H6″), 6.36 (d, *J* = 2.5 Hz, 1H, H4″), 6.61 (d, *J* = 8.5 Hz, 1H, H7″), 7.37 (d, *J* = 8.4 Hz, 2H, H2′ & H6′), 7.43–7.48 (m, 1H, H4‴), 7.48–7.53 (m, 2H, H3‴ & H5‴), 7.86 (d, *J* = 8.4 Hz, 2H, H3′ & H5′), 7.88–7.92 (m, 2H, H2‴ & H6‴) ppm.

^*13*^ *C‐NMR* (*101 MHz, CDCl*_*3*_) *δ* = 34.9 (t, C5), 42.7 (d, C3), 45.3 (d, C4), 46.9 (t, C6), 50.2 (t, C2), 69.6 (t, ‐O‐CH_2_‐), 98.1 (d, C4″), 101.2 (t, C2″), 105.7 (d, C6″), 108.0 (d, C7″), 122.9 (d, 2C, C2‴ & C6‴), 123.3 (d, 2C, H3′ & H5′), 128.3 (d, 2C, H2′ & H6′), 129.2 (d, 2C, C3‴ & C5‴), 131.0 (d, C4‴), 141.7 (s, C7a″), 147.6 (s, C1′), 148.3 (s, C3a″), 151.7 (s, C4′), 152.8 (s, C1‴), 154.5 (s, C5″) ppm.
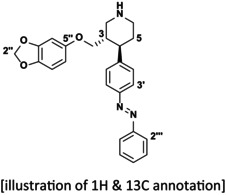


### 2.2 UV–Vis Spectroscopy

UV–Vis measurements were conducted on a UV 1800 UV/Vis spectrophotometer from Shimadzu. Spectra were recorded in a range from 265 to 600 nm with incremental steps of 0.5 nm and fast settings. Spectra were recorded in triplicate to spot potential instabilities. Measurements in DMSO were conducted at a concentration of 50 μM.

Spectra were recorded right after preparation of the sample (completely relaxed = (*E*)‐isomer) and after irradiation with light of 365 and 400 nm. OmniCure LED heads of 365 and 400 nm were used for irradiation set to 100% power (OmniCure LX400, max. power 320 mW). The samples were typically irradiated for 5 s to reach the photostationary state (PSS).

### 2.3 Materials

Cell culture supplies were obtained from Sigma‐Aldrich (St. Louis, MO, USA). [^3^H]5‐HT ([^3^H]5‐hydroxytryptamine; serotonin; 28.1 Ci/mmol) was purchased from PerkinElmer, Boston, MA. [^3^H]MPP^+^ (85 Ci/mmol) was supplied by American Radiolabeled Chemicals (St Louis, MO, USA). Serotonin (5‐HT) and paroxetine were purchased from Sigma‐Aldrich (St. Louis, MO, USA). 1‐methyl‐4‐phenylpyridinium ion (MPP^+^) was purchased from Research Biochemicals International, Natick, MA. The Research Resource Identifiers (RRIDs) for YhDAT, YhSERT, and hNET are (from addgene.org): YhDAT—RRID:Addgene_90228; YhSERT—RRID:Addgene_70103; hNET—RRID:Addgene_15475.

### 2.4 Cell Culture

Human embryonic kidney 293 cells (HEK293 cells) stably expressing the human isoforms of YFP‐tagged DAT (YhDAT), SERT (YhSERT), and the rat isoform of YFP‐tagged GAT1 (YrGAT1) and untagged NET (hNET), as well as HEK293 cells transiently transfected with YFP‐tagged human GAT1 (YhGAT1) and untagged human GAT3 (hGAT3) were cultured in Dulbecco's modified Eagle's medium (DMEM) with high glucose (4.5 g/L) and L‐glutamine (584 mg/L), supplemented with 10% fetal calf serum (FCS), 100 units/mL penicillin, and 100 μg/mL streptomycin. The cells were cultured in a humidified atmosphere (37°C, 5% CO_2_) on standard plastic culture ware. To maintain the selection pressure for the stable cell lines, 150 units/mL Geneticin was added. Cells were typically not passaged over 25 times. For uptake inhibition experiments, cells were seeded at 3.6 × 104 cells/0.2 mL or 3.6 × 104 cells/well density into poly‐d‐lysine‐coated 96‐well plates 24 h prior to the experiment.

The HEK293 cell line is not listed as a commonly misidentified cell line by the International Cell Line Authentication Committee (ICLAC; http://iclac.org/databases/cross‐contaminations/). HEK293 cells have been authenticated using highly polymorphic short tandem repeat loci (STRs). STR loci were amplified using the PowerPlex 16 HS System (Promega). Fragment analysis was done on an ABI3730xl (Life Technologies), and the resulting data were analyzed with GeneMarker HID software (Softgenetics). The results were compared with the Cellosaurus database in terms of matching to the cell line HEK293‐DR‐GFP‐RAD51B‐9 (RRID:CVCL_XX01): parental HEK293 cells (96.7%), stable HEK293 cell lines expressing YhDAT (90%), hNET (100%), YhGAT1 (93.8%) and YhSERT (96.7%).

### 2.5 Uptake Inhibition Experiments

For uptake inhibition experiments, the cell medium was aspirated, and the cells were washed once with 0.2 mL/well Krebs‐HEPES buffer (KHB) at room temperature (KHB composition: 10 mM HEPES, 120 mM NaCl, 3 mM KCl, 2 mM CaCl_2_, 2 mM MgCl_2_, and 20 mM glucose, pH adjusted to 7.3–7.4) as described (Ilic et al. 2020). The cells were preincubated with increasing concentrations of the substance of interest, diluted in KHB (0.05 mL/well) while irradiating the cells with UV light from below (365 nm/20 V/0,23A) using light‐emitting diodes or leaving the cells without additional light treatment for comparison. After 10 min, the preincubation solution was replaced by 0.05 mL/well KHB still containing the substance of interest and tritiated substrate additionally (50 nM [^3^H]MPP^+^ for the YhDAT; 100 nM [^3^H]5HT for the YhSERT; 30 nM [^3^H]GABA for the YhGAT1, hGAT3 and YrGAT1 and 20 nM [^3^H]MPP^+^ for the hNET) maintaining UV light treatment/no additional light treatment. Substrate uptake was stopped after 1 min (for [^3^H]5HT) or 3 min (for [^3^H]GABA and [^3^H]MPP^+^), respectively, by exchange of the substrate‐containing buffer with 0.2 mL/well ice‐cold KHB. The KHB was aspirated immediately, and the cells were lysed in 0.2 mL 1% SDS, transferred to 6‐mL scintillation vials, and assayed for [^3^H] content by liquid scintillation counting. Non‐specific uptake was determined in the presence of 50 μM GBR‐12909 (for YhDAT); 30 μM paroxetine (for YhSERT); 100 μM nisoxetine (for hNET); 1 mM tiagabine (for YhGAT1 and YrGAT1); and 100 μM (S)‐SNAP‐5114 (for hGAT3).

### 2.6 Whole‐Cell Patch‐Clamp Recordings

Whole‐cell patch‐clamp recordings were performed as recently described (Niello et al. 2023). HEK293 cells stably expressing wild‐type GFP‐tagged human SERT were seeded at low density on poly‐D‐lysine‐coated dishes. Twenty hours after seeding, these cells were subjected to patch‐clamp recordings in the whole‐cell configuration. The cells were continuously superfused with an external solution containing 140 mM NaCl, 3 mM KCl, 2.5 mM CaCl_2_, 2 mM MgCl_2_, 20 mM glucose, and 10 mM HEPES (pH adjusted to 7.4 with NaOH) and azo‐paroxetine in its active (*Z*) and inactive (*E*) configurations, respectively, were diluted therein. The internal solution in the patch pipette contained 133 mM potassium gluconate (CH_2_OH(CHOH)_4_COOK), 5.9 mM NaCl, 1 mM CaCl_2_, 0.7 mM MgCl_2_, 10 mM HEPES, 10 mM EGTA (pH adjusted to 7.2 with KOH). Drugs were applied using a 4‐tube ALA perfusion manifold (NPI Electronic GmbH, Germany) and a DAD‐12 superfusion system (Adams & List, Westbury, NY, USA) allowing for complete solution exchange around the cells within 100 ms. Currents were recorded via an Axopatch 200B amplifier (MDS Analytical Technologies, Sunnyvale, California) and analyzed using Clampfit 10.2 software. Passive holding currents were subtracted, and the traces were filtered using a 100 Hz digital Gaussian low‐pass filter.

### 2.7 Data Analysis

Data from uptake experiments were subjected to non‐linear, least squares curve fitting to equations for a rectangular hyperbola using a Marquardt–Levenberg algorithm (GraphPad Prism v.9). The fit was not improved by employing a logistic equation (Hill equation).

#### 2.7.1 Modeling

All studies have been performed in the Schrödinger 18‐3 software package (Farid et al. 2006; Sherman, Beard, et al. 2006; Sherman, Day, et al. 2006).

### 2.8 Induced‐Fit Docking

The protein structures for the human SERT (PDB ID 5I6X, Na2 ion from PDB ID 5I71, Coleman et al. 2016) (the mutated residue S439 was changed back to the native residue T439) were prepared with the Schrödinger Preparation Wizard (LigPrep, Schrödinger LLC, New York, NY, 2018), and the ligands were prepared with Schrödinger Ligprep (LigPrep, Schrödinger LLC, New York, NY, 2018), all with default settings. The protonation states of the ligands were additionally checked with chemicalize (https://chemicalize.com, pKa of the nitrogen in the piperidine ring azo‐paroxetine: 9.75, 99.6% protonated at pH 7.4). The induced‐fit (Farid et al. 2006; Sherman, Beard, et al. 2006; Sherman, Day, et al. 2006) was carried out with an extended sampling protocol (generates up to 80 poses using automated docking settings) and with an implicit membrane (generated from PDB ID 5I6X in the OPM database; Lomize et al. 2012). All other options were kept as default (force field OPLS3e, no constraints, ligand conformational sampling within 2.5 kcal/mol, softened potential (van der Waals radii scaling), prime refinement of residues within 5 Å of ligand poses, glide redocking within 30 kcal/mol of lowest‐energy structure). The orthosteric binding site was defined by all residues within 4.5 Å distance of paroxetine in PDB ID 5I6X (residue selection: Y95, A96, D98, A169, I172, A173, Y175, Y176, F335, S336, G338, F341, S438, T439, G442, L443, T497, and V501).

### 2.9 Docking Pose Analysis

The generated poses were hierarchically clustered by average linkage according to the volume overlap of the common scaffold (= heavy atoms of paroxetine substructure, Maestro tool volume overlap; Farid et al. 2006; Sherman, Beard, et al. 2006; Sherman, Day, et al. 2006). A volume score matrix was computed and the total number of clusters (14) was generated according to the Kelley function minimum (Kelley et al. 1996). Poses of the most populated cluster (cluster 12, 54 poses in total, 34 (*Z*)‐azo‐paroxetine and 20 (*E*)‐azo‐paroxetine poses, see Figures S1–S4 in Section A IV.) were analyzed according to their emodel scores and glide gscores. The boxplots in the supplement were computed in R 3.4.3 with the package ggplo2 3.2.0.

### 2.10 MM‐GBSA (Molecular Mechanics Generalized Born Surface Area) Calculations

The calculations for the most promising docking poses were performed with the Schrödinger Prime Energy Calculation (Prime, Schrödinger LLC, New York, NY, 2018) module using the OPLS3e force field. The protein–ligand complexes were first refined using a VSGB (= variable‐dielectric generalized Born model, considering residue‐dependent effects) water model and applying an implicit membrane (implementing a low‐dielectric to consider the hydrophobic membrane environment, coordinates taken from OPM structure PDB ID 5I6X; Coleman et al. 2016; Lomize et al. 2012) with a local optimization sampling algorithm (ligand + residues within 5 Å). After refinement, a molecular mechanics energy calculation was performed, including the same solvation and membrane models and a minimization sampling method to calculate the binding energies of the protein–ligand complexes.

## 3 Results

**FIGURE 1:**
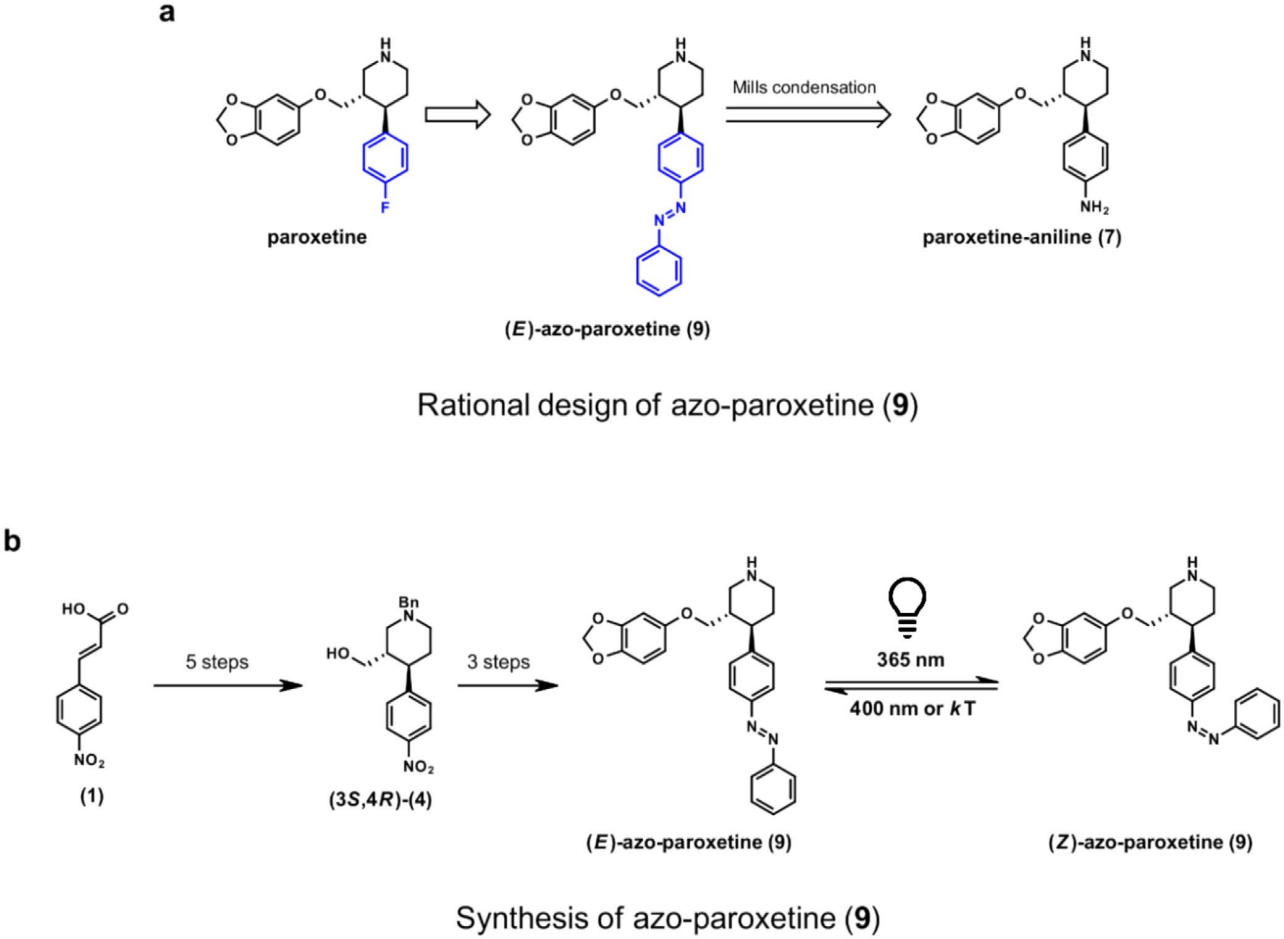
(a) Retrosynthetic approach and rational design toward target azo‐paroxetine (**9**), (b) Synthesis of azo‐paroxetine (**9**). Reagents and conditions for steps 1–5 (a–e) and 6–8 (f–h): a) 1) (COCl)_2_, DMF (cat.), DCM, rt., 1 h; 2) benzylamine, NEt_3_, DCM, rt., 16 h, 93% (b) methyl acrylate, TBSOTf, NEt_3_, t‐BuOH, DCE, rt., 20 h, 36% cis‐product, 33% trans‐product (c) epimerization of the cis‐piperidone: NaOMe, MeOH, rt., 5 min, 68% (d) 1) NaBH_4_, BF_3_•OEt_2_, THF, 0°C—50°C, 15 h; 2) MeOH, reflux, 1.5 h, 93% (e) vinyl acetate, Amano Lipase, MTBE, rt., 47 h, 33%, 99% ee (f) 1) MsCl, NEt_3_, DCM, 0°C—rt., 10 min; 2) sesamol, NaH, DMF, 90°C, 2 h, 63% (g) H_2_, Pd/C, MeOH/EtOAc (4/1), 60°C, 16 h, 84% h) nitrosobenzene, AcOH, rt., 16 h, 60%.

We established azo‐paroxetine (**9**) retrosynthetically from an amino analog of paroxetine via a Mills condensation with nitrosobenzene (Figure 1a). We obtained the aniline (**7**) from a suitable nitro precursor for the placement of the aniline fragment similar to the literature on paroxetine synthesis (Takasu et al. 2005), commencing with a suitable nitro starting material instead of the fluoro version. 4‐Nitrocinnamic acid (**1**) was transformed into the corresponding amide using benzyl amine. The 𝝰‐unsaturated amide underwent cyclization with methyl acrylate via a double Michael addition. The undesired *cis*‐piperidone was epimerized to obtain larger quantities of the desired *trans*‐product. In the next step, the ester function and the lactam were both reduced using NaBH_4_ and BF_3_ in refluxing THF (Norris et al. 2000), leaving the nitro group intact. The racemic alcohol was used to attempt an enzymatic kinetic resolution via Lipase‐catalyzed acetylation (de Gonzalo et al. 2001). An initial screening identified Amano Lipase PS from *Burkholderia cepacia* as a suitable bio‐catalyst. The less reactive alcohol was found to have the desired (3*S*,4*R*)‐configuration of paroxetine (details see Supporting Information). The (3*S*,4*R*)‐alcohol was successfully isolated with an ee of 99%. Subsequent etherification with sesamol and reduction with hydrogen and Pd(C) afforded the desired aniline analog of paroxetine by incorporating the original phenyl ring right into the photoswitchable structure. Finally, azo‐paroxetine (**9**) resulted from condensation with nitrosobenzene (Figure 1a).

**FIGURE 2:**
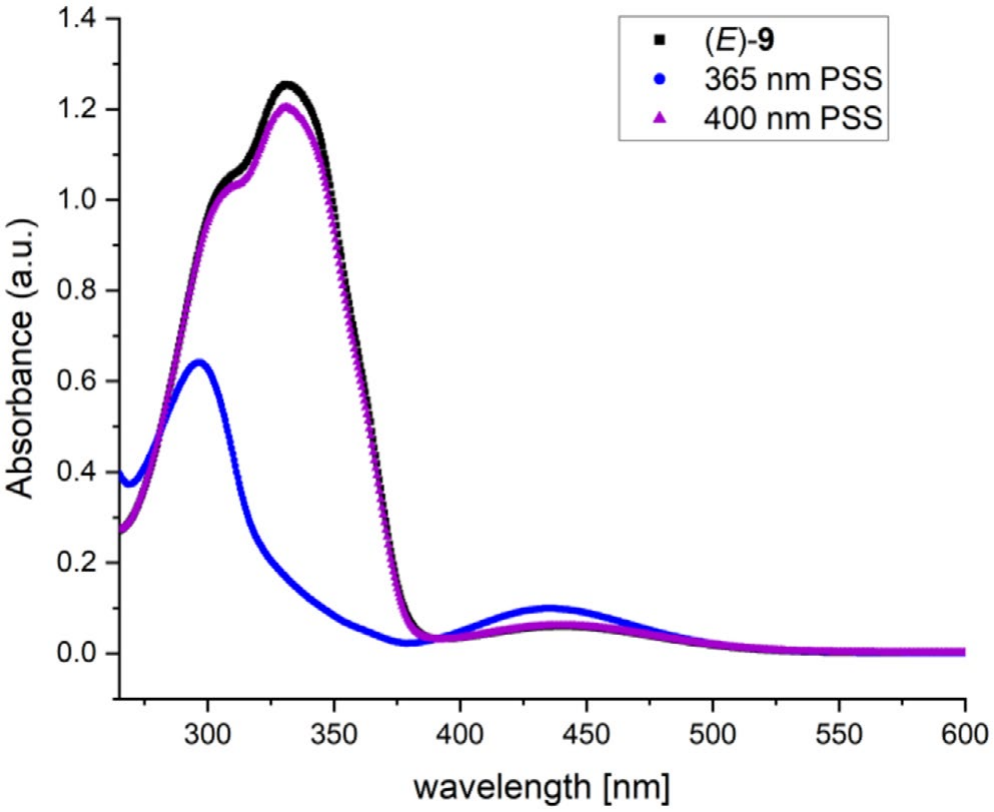
UV–Vis spectra of (*E*)‐azo‐paroxetine (**9**) 50 μM in DMSO (in the dark, black curve), and it's photostationary states (PSS) after irradiation at 365 nm ((*E*) to (*Z*) isomerization, blue curve) and after irradiation at 400 nm ((*Z*) to (*E*) isomerization, purple curve).

After synthesis, we first characterized the photophysical properties of azo‐paroxetine (**9**). The spectrum of dark‐adapted azo‐paroxetine (**9**) (the *E*‐state) exhibited the features expected for a *trans*‐ or (*E*)‐azo‐derivative. Upon illumination with blue light (intensity 600 mW/cm^2^ 10 mm focusing lens, λ 365 nm), a marked change was observed indicative of (*E*)−/(*Z*)‐isomerization (Figure 2).

**FIGURE 3:**
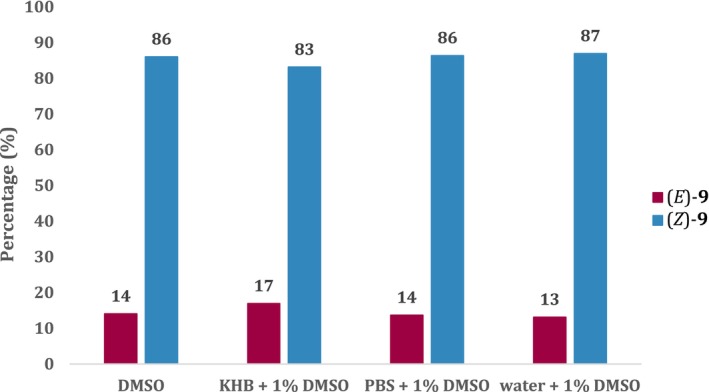
Quantification results of (*E*)‐ and (*Z*)‐isomer content in photostationary states of azo‐paroxetine (**9**) after irradiation with 365 nm in several solvents and solvent mixtures. Switching concentration 10 μM, concentration in UHPLC‐TOF‐MS sample 1 μM, UHPLC‐TOF‐MS sample preparation with water/acetonitrile 90/10.

Additionally, quantification measurements with UHPLC‐TOF‐MS were conducted to determine the efficiency of the (*E*) to (*Z*) isomerization of azo‐paroxetine. Ten micrometers azo‐paroxetine (**9**) samples were prepared in four different solvents to investigate differences in the switching behavior between DMSO and aqueous systems. Moreover, biological uptake inhibition assay conditions were mimicked during the switching experiments by choosing respective solvents and sample concentrations. Samples representing the dark state were taken before irradiation, samples representing the (*Z*)‐state were obtained after irradiation with blue light (intensity 600 mW/cm^2^ 10 mm focusing lens, λ 365 nm) for 30 s. Within this study, we observed very good switching behavior for azo‐paroxetine (**9**) in comparison with other azobenzene scaffolds (usually ~80% (*Z*)‐compound; Bandara and Burdette 2012). Photostationary states of 83%–87% (*Z*)‐isomer were reached upon irradiation (Figure 3). Such high switchability is crucial for distinct differences in biological uptake inhibition assays between the irradiated and non‐irradiated samples (*vide infra*).

**FIGURE 4:**
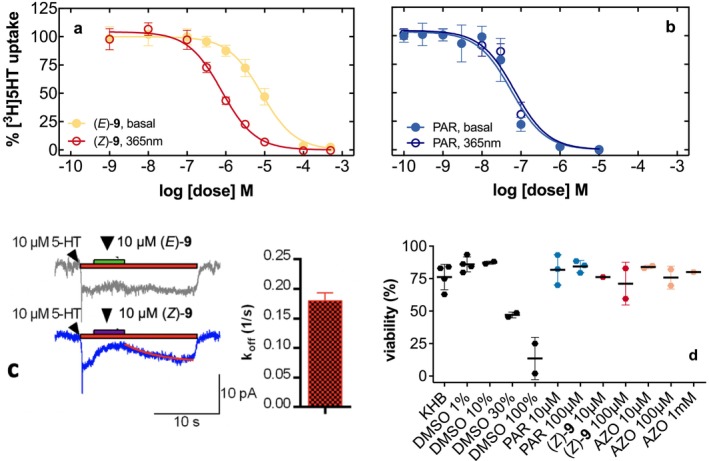
Uptake inhibition experiments using azo‐paroxetine (**9**) or paroxetine in a dose‐dependent manner at stable SERT‐expressing HEK293‐cells (panels a and b). The symbols represent three to four experiments performed in triplicate on different days, the error bars denote standard deviation. The red curves indicate the conditions promoting the optical switching (at a wavelength of 365 nm), the black curves indicate standard illumination. Panel c: Electrophysiological assessment of azo‐paroxetine (**9**) (10 μM) after standard light treatment (upper part; (*E*)‐isomer) and optical switching (lower part; (*Z*)‐isomer, PSS 365 nm). Currents were induced using the substrate 5‐HT (at a concentration of 10 μM, duration indicated by red bar) and afterward, azo‐paroxetine was included for the time indicated by the additional bar. The rate of current recovery upon removal of (*Z*)‐**9** from the bath solution (i.e., *k*_off_) was estimated by a fit of a monoexponential function to the current trace (see solid red line). The inset shows the summary of the off‐rates determined from experiments, such as the one shown in the lower panel of c (*n* = 5). Panel d: Viability assay employing trypan blue. As a positive control, we employed DMSO at increasing concentrations and then examined azo‐paroxetine (**9**) in a dose‐dependent manner. PAR = paroxetine.

Next, we tested whether the (*E*)‐isomer and the photoequilibrated (*E*)−/(*Z*)‐mixture (also referred to as (*Z*)‐**9** in the publication) of azo‐paroxetine (**9**) were able to interact with human SERT stably expressed in human embryonic kidney 293 (HEK293) cells. Whereas both states (dark and PSS at 365 nm) of compound (**9**) inhibited SERT‐mediated uptake of the tritiated substrate serotonin in a concentration‐dependent manner, there was a marked 12‐fold difference between (*E*)‐isomer and the PSS at 365 nm of azo‐paroxetine (**9**) (Figure 4; IC_50(E)_: 9.23 μM, IC_50(365nm)_: 0.76 μM). In contrast, light stimulation of the parent compound paroxetine had no impact on its pharmacological properties as comparable IC_50_ values to the non‐illuminated sample were obtained (IC_50:365nm_: 70 ± 0.03 nM and IC_50:NL_: 54 ± 0.01 nM for 365 nm and absence of light, respectively; Figure 4).

To further examine the molecular details of the interaction of azo‐paroxetine (**9**) with SERT, we employed electrophysiological recordings. We recorded inwardly directed currents elicited by serotonin in a patch‐clamp setup in the whole‐cell configuration to study the kinetic interaction profile of azo‐paroxetine (**9**) at SERT (Figure 4c). Addition of photoequilibrated (365 nm) azo‐paroxetine (**9**) completely abolished the inwardly directed current (10 μM; Figure 4c, blue trace). The off‐rate determined for photoequilibrated (365 nm) azo‐paroxetine (**9**) (0.18 ± 0.031; *n* = 5) was in the range of other SERT blockers, but it was much higher than that of the unmodified paroxetine (~0.002 s^−1^; Plenge et al. 2007). In stark contrast, (*E*)‐azo‐paroxetine (**9**) (10 μM) induced marginal blockade of the serotonin‐induced currents. However, application of higher concentrations (100 μM) impeded further measurement as the seal of the patch pipette was consistently lost. We speculated that (*E*)‐azo‐paroxetine (**9**) might exert toxic effects at higher concentrations, as described earlier for paroxetine (Schuster et al. 2007). Therefore, we recorded cellular toxicity effects; however, only DMSO had an effect at higher concentrations, but neither azo‐paroxetine (**9**) nor azobenzene (Figure 4d). Nonetheless, due to the low blocking ability of (*E*)‐azo‐paroxetine (**9**), we were not able to determine the off‐rate of this compound in electrophysiological recordings (Figure 4c).

**FIGURE 5:**
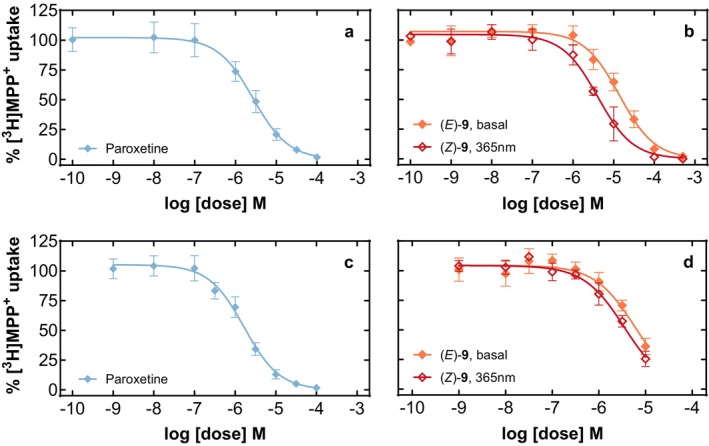
Uptake inhibition experiments using paroxetine or azo‐paroxetine (**9**) in a dose‐dependent manner at stable NET‐expressing HEK293‐cells (Panels a and b) and DAT‐expressing HEK293‐cells (Panels c and d), as indicated. The symbols represent 3–4 experiments performed in triplicate on different days, the error bars denote standard deviation. The red curves indicate the conditions promoting the optical switching (at a wavelength of 365 nm), the black curves indicate standard illumination.

We reasoned that the addition of an azo‐moiety to paroxetine might possibly change the selectivity pattern of the initial compound. Hence, we examined whether azo‐paroxetine (**9**) interacted with other members of the monoamine transporter family. In keeping with the reported paroxetine selectivity for SERT (Owens et al. 2001), we observed 30 and 50 fold higher IC_50_ values at DAT and NET, respectively (Table 1). In contrast, the interactions of azo‐paroxetine (**9**) at DAT and NET were comparable to the inhibitory potency observed at SERT (Figure 5 and Table 1).

**TABLE 1:** Inhibitory profiles of paroxetine and azo‐paroxetine (**9**) at SERT, DAT, and NET, either under standard light conditions or at the wavelength inducing the optical switching of azo‐paroxetine (**9**). Values denote the results of 3–4 experiments performed in triplicate ± SD (nd, not determined).

Substance	*λ* (nm)	SERT IC_50_ ± SD (μM)	*n*	NET IC_50_ ± SD (μM)	*n*	DAT IC_50_ ± SD (μM)	*n*
Paroxetine	Basal	0.054 ± 0.014	4	2.76 ± 0.65	3	1.71 ± 0.27	4
Paroxetine	365	0.070 ± 0.026	4	n.d.		n.d.	
Azo‐paroxetine (**9**)	Basal	9.23 ± 3.06	3	15.4 ± 4.04	4	6.33 ± 2.06	3
Azo‐paroxetine (**9**)	365	0.76 ± 0.18	3	5.41 ± 3.32	3	3.77 ± 0.79	3

At SERT, we found that light stimulation resulted in a marked leftward shift in the concentration‐response curve with a 12‐fold reduction in the corresponding IC_50_ value (Table 1). When compared to these values, the interaction between the (*E*)‐isomer and photoequilibrated (365 nm) state of azo‐paroxetine (**9**) and human DAT and NET was significantly less pronounced (Table 1). However, the optopharmacological switching increased the affinity toward NET as we observed a ~threefold reduction in the IC_50_ value. At DAT, the effect of optical switching on inhibitory potency was even smaller (1.7‐fold difference). Upon visual inspection of the uptake inhibition curves and also when looking at the relatively low sequence identity of the two transporters, this difference seems to be rather negligible.

Interestingly, there was a relatively mild loss in affinity of azo‐paroxetine (**9**) in comparison with the parent compound paroxetine at DAT and NET (only a loss by a factor of ~2 to 5 while the loss in affinity at SERT was at a factor of 171); thus, a relative improvement in affinity by the addition of azobenzene.

**FIGURE 6:**
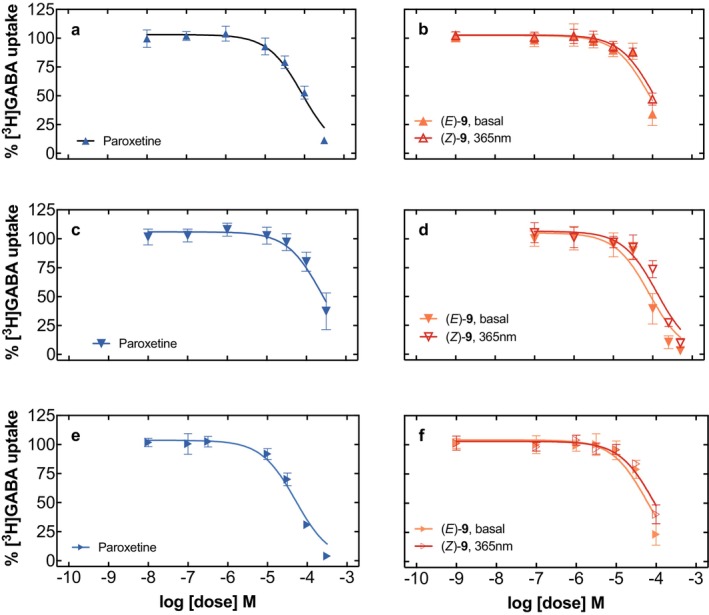
Uptake inhibition assays at various GABA transporters: Human GAT1 (Panel a, paroxetine; Panel b, azo‐paroxetine (**9**)), rat GAT1 (Panel c, paroxetine; Panel d, azo‐paroxetine (**9**)), human GAT3 (Panel e, paroxetine; Panel f, azo‐paroxetine (**9**)). The symbols represent 3–4 experiments performed in triplicate on different days, the error bars denote SD. The red curves indicate the conditions promoting the optical switching (at a wavelength of 365 nm), the black curves indicate standard illumination.

The observation that merging paroxetine with the photoswitchable moiety azobenzene reduced its selectivity at SERT when compared to DAT and NET prompted us to investigate the interaction profile at more distantly related transporters. First, we chose to examine another SLC6 family member—the transporter for gamma‐aminobutyric acid (GABA; GAT1), which is a target of the antiepileptic drug tiagabine (Bhatt et al. 2023; Jurik et al. 2015; Motiwala et al. 2022). [^3^H]GABA uptake in HEK293‐cells stably expressing human GAT1 was inhibited by paroxetine in a concentration‐dependent manner, yielding an IC_50_ value of more than 120 μM (Figure 6a; Table 2). Azo‐paroxetine (**9**) inhibited GAT1 to a comparable extent and without difference between the (*E*)‐isomer and photoequilibrated (365 nm) state (Figure 6b). Next, we examined whether the same holds true for a different mammalian species; we tested the rat GAT1 and found an even lower affinity of paroxetine at a value of around 250 μM (Figure 6c; Table 2). Here, the addition of azobenzene (**9**) improved the affinity around two to three‐fold; interestingly, however, a lower affinity was observed with higher (*Z*)‐isomer content (Figure 6d; Table 2). Finally, we included the human GAT3 to extend our experiments to another SLC6 transporter from the GABA transporter family (Kickinger et al. 2019). Again, GABA uptake was inhibited both by paroxetine and azo‐paroxetine (**9**), with a slight loss in affinity visible in the increased (*Z*)‐isomer state of the latter compound (Figure 6e,f; Table 2). We concluded from these experiments on GAT‐mediated uptake that (i) paroxetine has—at best—only low affinity to the GABA transporters included in this study and (ii) the addition of azobenzene mildly enhances this affinity.

**TABLE 2:** Inhibitory profile of paroxetine and azo‐paroxetine (**9**) at GAT1 (human and rat species) and human GAT3, either under standard light conditions or at the wavelength inducing the optical switching of azo‐paroxetine (**9**). Values denote the results of three to four experiments as indicated, performed in triplicate ± SD. nd, not determined.

Substance	*λ* (nm)	rGAT IC_50_ ± SD (μM)	*n*	hGAT1 IC_50_ ± SD (μM)	*n*	hGAT3 IC_50_ ± SD (μM)	*n*
Paroxetine	Basal	264 ± 140	4	122 ± 81	4	51 ± 3.7	3
Paroxetine	365	nd		nd		nd	
Azo‐paroxetine (**9**)	Basal	88.9 ± 11.8	4	80.8 ± 21.6	3	55 ± 16.4	3
Azo‐paroxetine (**9**)	365	126 ± 20.6	3	107 ± 15	3	90.5 ± 24.4	3

**FIGURE 7:**
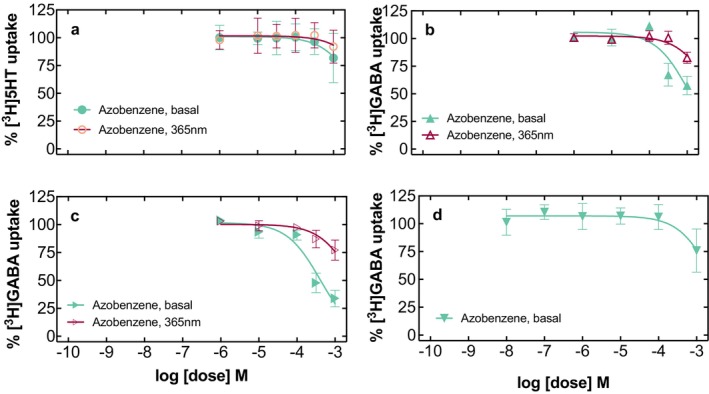
Functional data on the interaction of azobenzene with HEK293‐cells stably expressing human SERT (Panel a), human GAT1 (Panel b), human GAT3 (Panel c), or rat GAT1 (Panel d). Shown are uptake inhibition curves of the individual tritiated substrates (indicated in the *Y*‐axis description) in the presence of azobenzene at increasing concentrations. The symbols represent three to four experiments performed in triplicate on different days; the error bars denote SD. The curves indicate the conditions promoting the optical switching (at a wavelength of 365 nm; open symbols), the curves with filled symbols indicate standard (basal) illumination.

Finally, intrigued by the increase in affinity at rat GAT1, we tested whether the azobenzene moiety had any affinity on its own at human SERT, at rat and human GAT1, and human GAT3. We examined concentrations of up to 1 mM first at human SERT and could not really observe any significant inhibitory effects even at the highest concentrations tested, and without any effect of excitation at 365 nm (Figure 7a). GABA transporters were slightly more reactive toward the administration of azobenzene, and in the (*E*)‐configuration, azobenzene was able to inhibit up to 40% of GABA uptake (Figure 7b). Human GAT3, however, was affected at even higher levels, and HEK293‐cells stably expressing GAT3 displayed inhibition of more than 60% (Figure 7c). When examining GAT1 of the rat species (Figure 7d), the value of (*E*)‐azobenzene (**9**) was comparable to the value of the human GAT1 (Figure 7b).

### 3.1 Molecular Docking of (*E*)‐ and (*Z*)‐azo‐Paroxetine in the Human SERT

**FIGURE 8:**
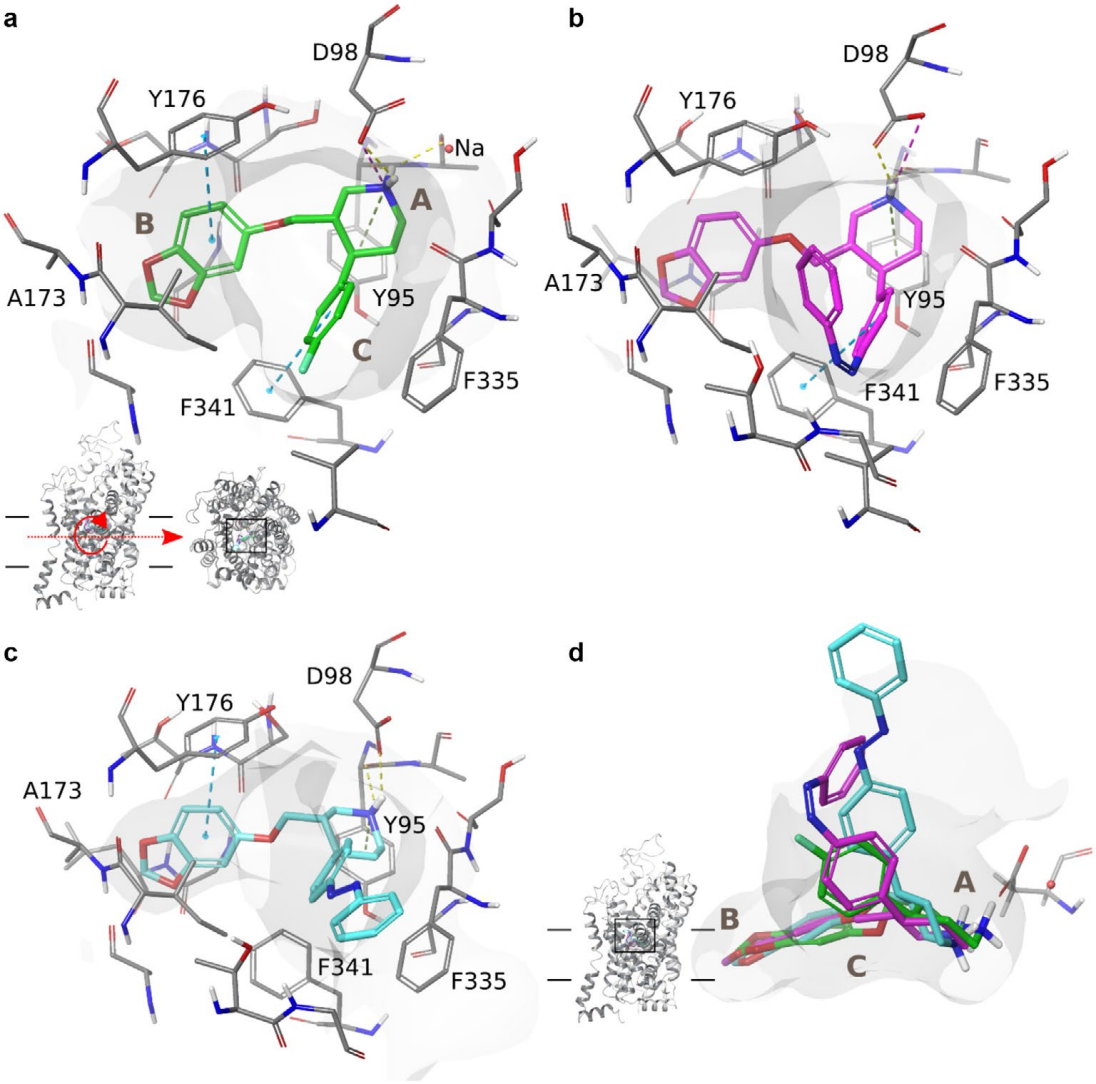
Panel a: Binding pose of paroxetine in the orthosteric site of the human SERT crystal structure 5I6X (Coleman et al. 2016). The letters A, B, and C indicate the different subpockets. Panel b: Selected binding pose of (*Z*)‐azo‐paroxetine (**9**) in the orthosteric site of human SERT. Panel c: Selected binding pose of (*E*)‐azo‐paroxetine (**9**) in the orthosteric site of human SERT. Panel d: Comparison of paroxetine (green), (*Z*)‐azo‐paroxetine (**9**) (pink), and (*E*)‐azo‐paroxetine (**9**) (cyan) in human SERT.

In order to investigate the molecular determinants that govern the activity difference of (*E*)‐ and (*Z*)‐azo‐paroxetine (**9**), we performed molecular docking studies in the orthosteric pocket of the human SERT crystal structure PDB ID 5I6X (Coleman et al. 2016). This structure was chosen because it was co‐crystallized with paroxetine and resolved at a higher resolution than the subsequently published structure 6AWN (3.14 and 3.62 Å, respectively) (Coleman and Gouaux 2018). By induced‐fit docking in the Schrödinger Suite 2018‐3 (Farid et al. 2006; Sherman, Beard, et al. 2006; Sherman, Day, et al. 2006), we obtained 45 poses for (*Z*)‐azo‐paroxetine (**9**) and 38 for (*E*)‐azo‐paroxetine (**9**). The docking scores (glide gscore and glide emodel score (Friesner et al. 2004)) were in agreement with the biological data: the 19 top ranked poses were occupied exclusively by (*Z*)‐azo‐paroxetine (**9**) poses, and the calculated mean glide and emodel scores over all poses showed a clear trend toward more favorable docking scores for (*Z*)‐azo‐paroxetine (**9**) ((*Z*)‐azo‐paroxetine (**9**) mean glide gscore/emodel −10.33 ± 1.06/−111.643 ± 14.16, (*E*)‐azo‐paroxetine (**9**) −9.35 ± 0.75/101.07 ± 10.33, see Figure S1 in Section A IV.). The poses were further analyzed by common scaffold clustering of the shared paroxetine substructure and by calculating the RMSD compared with the co‐crystallized paroxetine in the human SERT structure 5I6X (Coleman et al. 2016). The most highly populated cluster (54 poses in total, 34 (*Z*)‐azo‐paroxetine (**9**) and 20 (*E*)‐azo‐paroxetine (**9**) poses) not only showed the smallest RMSD compared with the crystal structure (mean RMSD 1.8 ± 0.8, see Figure S2 in Section A IV.), but also showed again a clear trend of better docking scores for the Z‐configuration ((*Z*)‐azo‐paroxetine (**9**) mean gscore/emodel score 10.66 ± 0.98/−115.27 ± 14.23, (*E*)‐azo‐paroxetine (**9**) 9.41 ± 0.65/104.52 ± 11.47, see Figure S3 in Section A IV.). As the azo‐compounds contain the paroxetine scaffold, we expect that these compounds should adopt a similar binding mode as observed in the crystal structure. In the human SERT crystal structure 5I6X, paroxetine displays characteristic interactions: in subpocket A, the cationic nitrogen of paroxetine forms a salt bridge and a hydrogen bond with D98, as well as a cation‐pi interaction and a hydrogen bond with Y95 (see Figure 8a). The benzodioxol moiety is positioned in the hydrophobic ridge of subpocket B formed by A169, I172, A173, and Y176, and shows a pi‐pi interaction with the latter. The fluorophenyl ring in subpocket C points toward T497 and V501, and forms a pi‐pi interaction with F341.

The docking poses in the most populated cluster showed similar orientations of the molecular features of the paroxetine substructure while the attached azobenzene needs to expand into the extracellular vestibule (see Figure 8d). Noteworthy, (*Z*)‐azo‐paroxetine (**9**) can adopt a binding mode more similar to paroxetine in the crystal structure than (*E*)‐azo‐paroxetine (**9**) as exemplified by lower mean RMSD scores ((*Z*)‐azo‐paroxetine (**9**) 1.5 Å ± 0.7, (*E*)‐azo‐paroxetine (**9**) 2.2 Å ± 0.8) and a lower mean distance of the cationic nitrogen to D98 ((*Z*)azo‐paroxetine (**9**) 4.5 ± 1.4, (E)‐azo‐paroxetine (**9**) 5.4 ± 1.3, see Figure S4 in Section A IV.) in the most populated cluster. D98 forms a salt bridge with the cationic nitrogen which is known to be the main affinity driving interaction among the monoamine transporters (Sorensen et al. 2012). To identify the poses representing the most plausible binding hypotheses for (*E*)‐ and (*Z*)‐azo‐paroxetine (**9**) from the most populated cluster, we defined the following selection criteria: the selected poses are (i) highest ranked among the top 15 poses according to emodel docking score, (ii) highest ranked among the top 15 poses according to glide docking gscore, (iii) show an RMSD value ≤ 1.5 Å to the paroxetine scaffold in the crystal structure, and (iv) form a salt bridge between the cationic nitrogen of the ligand and the charged side chain oxygen of D98 (distance ≤ 4.5 Å). For (*Z*)‐azo‐paroxetine (**9**), we extracted six poses that fulfill all criteria (see Table S1 in Section A IV.). Interestingly, for (*E*)‐azo‐paroxetine (**9**) there is not a single pose fulfilling all criteria. Therefore, we extracted four poses which fulfill three out of the four criteria (see Table S1 in Section A IV.). To finally select the most promising poses, we performed MM‐GBSA calculations (Farid et al. 2006; Sherman, Beard, et al. 2006; Sherman, Day, et al. 2006) to rank the selected poses according to their estimated binding free energy. All selected six (*Z*)‐azo‐paroxetine (**9**) poses showed a more favorable estimated binding free energy than the selected four (*E*)‐poses (see Table S1 in Section A IV.). The top scored poses are depicted in Figure 8b–d. (*Z*)‐azo‐paroxetine (**9**) could undergo the same interactions as paroxetine in the crystal structure while accommodating the attached azobenzene moiety in the extracellular vestibule. In contrast, the (*E*)‐azo‐paroxetine (**9**) piperidine moiety had to turn slightly toward the extracellular vestibule to allow the accommodation of the azobenzene moiety in the extracellular vestibule, which resulted in increased distance to D98 (4.3 and 5.1 Å, respectively) and in the loss of interaction with F341.

## 4 Discussion

SERT belongs to the monoamine NTTs, regulating the extracellular concentration of serotonin and thereby maintaining serotonergic neurotransmission. Due to dysfunctions of the serotonergic system associated with severe neuropsychiatric disorders like depression or anxiety, SERT represents an important target for both clinically relevant drugs like antidepressants and anxiolytic medicines, but also recreational drugs (Kristensen et al. 2011). As currently marketed drugs often exhibit adverse effects, new drugs with higher SERT selectivity are needed. Photopharmacology allows highly precise temporal and spatial control of bioactive compounds and therefore shows great potential in significantly decreasing adverse side effects in drugs (Lerch et al. 2016).

Our study reports on the development of the novel photoswitchable azo‐paroxetine (**9**) and its pharmacological interaction profile at SERT: similar to a previously reported azo‐citalopram (Cheng et al. 2020), our azo‐paroxetine (**9**) serves as a tool compound that can be used to control SERT activity in a precise and reversible manner. Moreover, we examined the interaction profile of azo‐paroxetine (**9**) with further members of the SLC6 transporter family like DAT, NET, GAT1, GAT3, and rat GAT1 to ascertain a possible loss in selectivity by the addition of the azobenzene moiety.

The synthesis of azo‐paroxetine (**9**) was accomplished in eight steps on the basis of the azo‐extension strategy. Therein, the photoswitchable moiety was incorporated into the new molecule by replacing the original fluoro substituent in paroxetine. The obtained azo‐paroxetine (**9**) was easily and reversibly switched between its (*E*)‐ and (*Z*) configurations and remained stable between these configurations. The respective spectrum of dark adapted azo‐paroxetine (**9**) exhibited the classical features expected for a *trans*‐ or (*E*)‐azo‐derivative. Quantifications of the (*E*)‐ to (*Z*) switchability showed that azo‐paroxetine (**9**) displayed good switching behavior by reaching photostationary states of 83%–87% (*Z*)‐isomer in different solvents upon irradiation. This was very satisfying, as high switchability is crucial for distinct activity differences of photoswitches at biological targets.

Performing uptake inhibition assays using standard procedures (Mayer et al. 2016) with HEK293 cells stably expressing SERT (Mayer et al. 2023); we found that the (*E*)‐isomer and the photoequilibrated (365 nm, 83% *Z*‐isomer content) state of azo‐paroxetine (**9**) displayed a 12‐fold difference in activity, with the (*Z*)‐isomer being the more potent configuration. This supports the potential of the compound as a useful tool compound in light‐induced modulation of SERT activity. Interestingly, similar values of up to a 12‐fold reduction in transport activity were determined in EAATs (Cheng et al. 2017). However, a related compound acting at SERT, azo‐citalopram, was found to result in a difference of around 45‐fold (Cheng et al. 2020).

In addition, we executed control experiments to ascertain that the parent compound paroxetine does not exhibit any changes in its pharmacological properties upon the same light exposure, which was indeed not the case (Figure 4a,b).

To further characterize the interaction profile of the compound with SERT, we examined the interaction of azo‐paroxetine (**9**) with SERT by means of electrophysiology. While the PSS at 365 nm with mainly (*Z*)‐configurated compound completely abolished currents induced by the physiological substrate serotonin, the compound in its (*E*)‐configuration was not able to inhibit those inwardly directed currents. The determination of the off‐rate for azo‐paroxetine (**9**) in mainly (*Z*)‐configuration revealed that it dissociates similarly to other SERT blockers but slower than the parent molecule. The application of higher concentrations of the compound in either configuration impeded further electrophysiological measurements due to interference with the giga‐seal of the patch pipette at the cells. Hence, we reasoned that higher concentrations of azo‐paroxetine may induce toxic effects which we subsequently assessed. However, we did not find any significant toxic impact on HEK293 cells up to high concentrations (Figure 4d). The findings that the addition of azobenzene to paroxetine reduced its selectivity at SERT over DAT and NET prompted us to examine possible off‐target activity of the compounds at other transporters of the SLC6 family.

Thus, we interrogated the interaction profiles with the human isoforms of DAT, NET, GAT1, and GAT3 and tested whether any species differences existed by utilizing rat GAT1; all transporters were stably expressed in previously (human DAT Niello et al. 2023; human NET: Rudin et al. 2022; rat GAT1: Sitte et al. 2002) or newly established HEK293 cell lines expressing human GAT isoforms (hGAT1, hGAT3). Our approach yielded no comparable pharmacological profile at all tested transporters and also no species differences between human and rat GAT1 transporters, which allowed us to conclude that compound (**9**) is highly specific for the intended use at the SERT, and also in the activated *Z*‐configuration. This is an important finding as it, for the first time, supports the notion that the addition of azobenzene groups does not hamper the underlying pharmacological activity of, by this approach, chemically modified pharmacologically active compounds per se. Interestingly, the addition of azobenzene to paroxetine decreased the affinity to SERT quite drastically (by 171 fold), while the already lower affinity of paroxetine to DAT was only reduced by a factor of ~3.7, and to NET by a factor of ~5. In addition, DAT and NET were the only transporters under scrutiny which also displayed an effect by azo‐paroxetine in its activated 365 nm equilibrated *Z*‐configuration; albeit it was rather a negligible effect of around 2 to 3 (Figure 5b,d).

As the azo‐compound (**9**) contains the paroxetine scaffold, a similar binding mode as observed in the crystal structure of paroxetine in SERT was to be expected (Coleman and Gouaux 2018; Coleman et al. 2016, 2020). Our docking results support this together with the overall good specificity of azo‐paroxetine (**9**) at SERT with the notion that the inactive *E*‐configuration has to adopt a slightly twisted configuration in the binding pocket in comparison with the active *Z*‐configuration and therefore most likely does not bind as tightly. Support for that notion is also lent by the electrophysiological characterization where stable interaction and thus inhibition of substrate‐induced current was only seen with the active (*Z*)‐azo‐paroxetine (**9**)—and a *k*_OFF_ value could be determined. It remains to be established whether the inactive (*E*)‐azo‐paroxetine (**9**) is dissociating in a fast manner from SERT.

Our results thus demonstrate that the activity of SERT can be quite exclusively manipulated by the novel optopharmacological agent azo‐paroxetine (**9**) in a reversible manner at a low probability of compound‐related off‐target effects. Hence, this lends support to the use of azobenzene moieties which appear not to be changing the pharmacodynamic activity of the conjugated compounds.

## Author Contributions

**Dominik Dreier:** investigation, conceptualization, writing – original draft. **Oliver John V. Belleza:** investigation, writing – review and editing. **Katharina Schlögl:** investigation, writing – original draft, writing – review and editing. **Stefanie Kickinger:** investigation, writing – review and editing. **Eva Hellsberg:** investigation, writing – review and editing. **Felix P. Mayer:** investigation, writing – review and editing. **Walter Sandtner:** investigation, writing – review and editing. **Philipp Mikšovsky:** investigation, writing – review and editing. **Matthias Schittmayer:** investigation, writing – review and editing. **Yuntao Hu:** investigation, writing – review and editing. **Kathrin Jäntsch:** investigation, writing – review and editing. **Marion Holy:** investigation, writing – review and editing. **Gerhard F. Ecker:** conceptualization, funding acquisition, writing – original draft. **Harald H. Sitte:** conceptualization, funding acquisition, writing – original draft. **Marko D. Mihovilovic:** conceptualization, funding acquisition, writing – original draft.

## Conflicts of Interest

H.S. is a Guest Editor for the “Transporters in the Nervous System” *Journal of Neurochemistry* Special Issue. The other authors declare no conflicts of interest.

## Supporting information


Data S1.

## Data Availability

General Notes on the chemical synthesis of compound (**9**) and intermediates, as well as synthetic procedures and analytical data obtained within this work are available in the Supporting Information. Additional information on the docking pose analyses is available in the Supporting Information. Any remaining information can be obtained from corresponding authors upon reasonable request.
