# Supplementary material for: Careful Examination of a Novel Azobenzene Paroxetine Derivative and Its Interactions With Biogenic Amine Transporters
Source: J Neurochem. 2025 Apr 24;169(4):e70068. doi: 10.1111/jnc.70068 (PMC12019583; doi:10.1111/jnc.70068)
Supplement: Supplementary file 1 — Data S1. [file JNC-169-0-s001.pdf]

Supplementary Material

A careful examination of a novel azobenzene derivate of paroxetine and its interactions with biogenic amine transporters (120/150 ch)

Dominik Dreier[1], Oliver V. Belleza[2], Katharina Schlögl[1], Stefanie Kickinger[3,&], Eva Hellsberg[3,%], Felix P. Mayer[2,$], Walter Sandtner[2], Philipp Mikšovsky[1], Matthias Schittmayer[4], Yuntao Hu[3], Kathrin Jäntsch[2], Marion Holy[2], Gerhard F. Ecker[3], Harald H. Sitte[2,§,*] and Marko D. Mihovilovic[1,§,*]

[1]Institute of Applied Synthetic Chemistry, TU Wien, Vienna, Austria

[2]Institute of Pharmacology, Center for Physiology and Pharmacology, Medical University of Vienna, Waehringerstr. 13a, A-1090 Vienna, Vienna, Austria

[3]Department of Pharmaceutical Chemistry, University of Vienna, Austria

[4]Institute for Chemical Technologies and Analytics, TU Wien, Vienna, Austria

[&]Present address: Department of Drug Design and Pharmacology, University of Copenhagen, Copenhagen, Denmark

[%]Present address: NIH (National Institutes of Health), NINDS (National Institute of Neurological Disorders and Stroke), BG 35A Convent Drive, Bethesda, MD 20892, USA

[$]Present address: Florida Atlantic University, The Brain Institute, 5353 Parkside Drive, MC17, RF RM 113, Jupiter, FL 33458, USA

[§]These authors contributed equally to the present manuscript

***Address correspondence to:**

Marko D. Mihovilovic, Institute of Applied Synthetic Chemistry, TU Wien, Vienna, Austria; Email: marko.mihovilovic@tuwien.ac.at

Harald H. Sitte, Institute of Pharmacology, Center for Physiology and Pharmacology, Medical University of Vienna, Vienna, Austria; Email: harald.sitte@meduniwien.ac.at

A I General Notes

Unless otherwise noted, chemicals were purchased from commercial suppliers and used without further purification.

NMR spectra were recorded on a Bruker *Avance Ultrashield 400* (^{1}H: 400 MHz, ^{13}C: 101 MHz). Chemical shifts are reported in ppm relative to the nominal residual solvent signal of $CDCl_3$ (^{1}H: 7.26 ppm, ^{13}C: 77.16 ppm).

For thin layer chromatography aluminium backed silica gel 60 F254 (Merck) was used.

Flash column chromatography (FC) was carried out with a Büchi Sepacore^{TM} MPLC system using silica gel 60 M (particle size 40-63 µm, 230-400 mesh ASTM, Macherey Nagel, Düren).

Melting points were determined by a Leica Galen III Kofler and a Büchi Melting Point B-545 and are uncorrected.

GC/MS spectra were measured on a Thermo Trace 1300 / ISQ LT (single quadrupole MS (EI)) using a standard capillary column BGB 5 (30 m x 0.25 mm ID).

An Agilent 6230 LC TOFMS mass spectrometer equipped with an Agilent Dual AJS ESI-Source was used for HR-MS analysis. The mass spectrometer was connected to a liquid chromatography system of the 1100/1200 series from Agilent Technologies, Palo Alto, CA, USA. The system consisted of a 1200SL binary gradient pump, a degasser, column thermostat, and an HTC PAL autosampler (CTC Analytics AG, Zwingen, Switzerland). A silica-based Phenomenex C-18 Security Guard Cartridge was used as stationary phase.

Data evaluation was performed using Agilent MassHunter Qulitative Analysis B.07.00. Identification was based on peaks obtained from extracted ion chromatograms (extraction width ± 20 ppm).

Mass spectrometry measurements for PSS determination were conducted on a Bruker timsTOF Pro operated in positive ion mode with ion mobility enabled. A Thermo Scientific Vanquish H UHPLC System was used as front end and compounds were separated in reversed phase mode on a Bruker Intensity Solo C18-2 column (2.1 mm x 100 mm, 1.8 µm particle size). Flow rate was kept constant at 400 µL/min, the following gradient was employed: 0 min: 2 % B; 3 min: 98 % B; 4 min: 98 % B, followed by 1 min reequilibration to 2 % B, total run time 5 min. Solvent A: H_2O, 0.1 % formic acid, Solvent B: Acetonitrile, 0.1 % formic acid. The software used for data acquisition was Hystar 5.1, otofControl 6.2 (Build 1.3) and Data Analysis 5.3.342. Data was analyzed using Skyline 21.1.0.278, quantification was performed at MS1 level.

Enantiomeric excess was determined *via* HPLC with a ChiralPak AS-H (250 mm x 4.6 mm ID) column on a Thermo Scientific/Dionex Ulitmate 3000 HPLC using mixtures of *n*-hexane/EtOH 0.5 - 7% over 45 minutes at 25 °C as mobile phase.

Specific rotation $[\alpha]_D^{20}$ was determined using an MCP 500 polarimeter from Anton Paar by the following equation: $[\alpha]_D^{20} = 100*\alpha/[c]*l$; c in [g/100 mL], l in [dm]

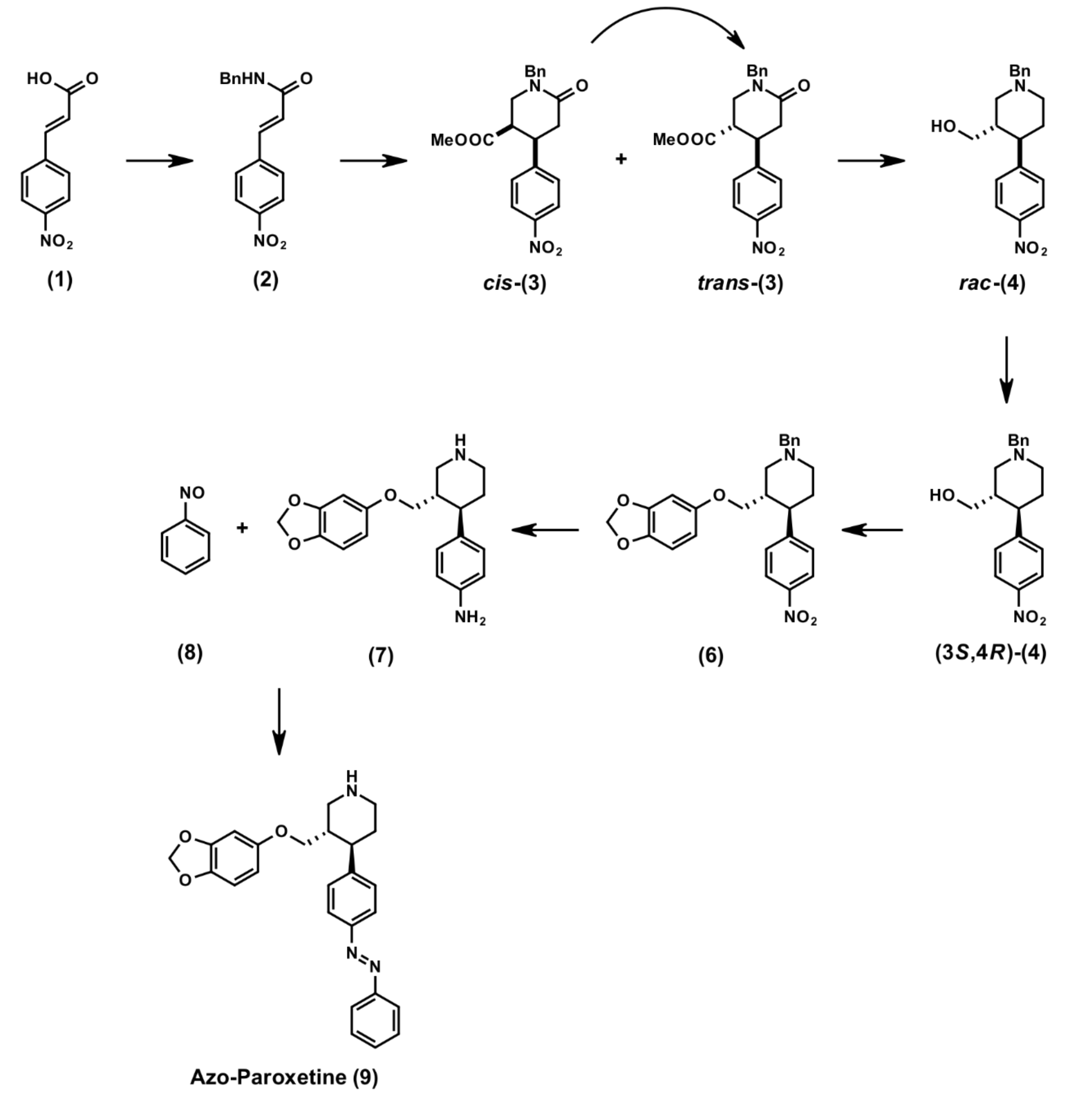

A II Synthetic Procedures

A II.1.1 *N*-Benzyl-3-(4-nitrophenyl)acrylamide (**2**)

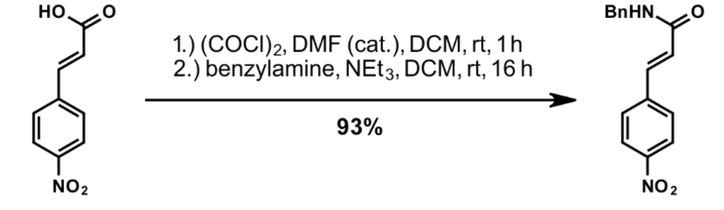

(1) 193.16 g/mol **(2)** 282.30 g/mol

4-Nitrocinnamic acid **(1)** (25.00 g, 129 mmol, 1 equiv.) was placed in a round bottom flask and the flask was evacuated and flushed with argon three times. Dry DCM (435 mL, 0.3 M relative to the starting material) and dry DMF (1 drop) were added *via* syringe. Oxalylchloride (33.3 mL, 388 mmol, 3 equiv.) was added dropwise under argon and the mixture was stirred at room temperature. After 1 hour TLC (sample quenched with MeOH) indicated full consumption of the starting material. The intermediate was obtained by removing the volatiles in *vacuo*. The isolated acid chloride was dissolved in dry DCM (425 mL) under argon. Benzylamine (13.87 g, 129 mmol, 1 equiv.) and NEt_3 (13.10 g, 129 mmol, 1 equiv.) were added while cooling with an ice bath, then the mixture was stirred at room temperature for 16 hours. The reaction was washed with 1 N HCl (400 mL) and 1 N NaOH (400 mL). Volatiles were removed in *vacuo* and the obtained crude material was purified by recrystallization from toluene to afford the pure product **(2)**.

**Yield**	93% (33.97 g, 120 mmol)
**Appearance**	pale yellow crystals
**Melting point**	189.5 – 190.0 °C (Lit.(Pardin et al., 2008): 188 – 190 °C)
**TLC-Analysis**	R_f = 0.71 (LP/EtOAc = 1/2)
**Sum formula**	$C_{16}H_{14}N_2O_3$
**GC-MS**	282 (17, M^+), 130 (29), 106 (100), 104 (32), 103 (29), 102 (83)

^{1}H-NMR (400 MHz, $CDCl_3$) δ = 4.60 (d, *J* = 5.7 Hz, 2H), 5.97 (br s, 1H), 6.53 (d, *J* = 15.6 Hz, 1H), 7.28 – 7.40 (m, 5H), 7.64 (d, *J* = 8.8 Hz, 2H), 7.72 (d, *J* = 15.6 Hz, 1H), 8.23 (d, *J* = 8.8 Hz, 2H) ppm.

^{13}C-NMR (101 MHz, $CDCl_3$) δ = 44.2 (t), 124.3 (d, 2C), 124.6 (d), 128.0 (d), 128.1 (d, 2C), 128.5 (d, 2C), 129.0 (d, 2C), 137.9 (s), 139.0 (d), 141.1 (s), 148.3 (s), 164.7 (s) ppm.

A II.1.2 Methyl (±)-*cis*-1-benzyl-4-(4-nitrophenyl)-6-oxopiperidine-3-carboxylate *cis*-(**3**) and

A II.1.3 Methyl (±)-*trans*-1-benzyl-4-(4-nitrophenyl)-6-oxopiperidine-3-carboxylate *trans*-(**3**)

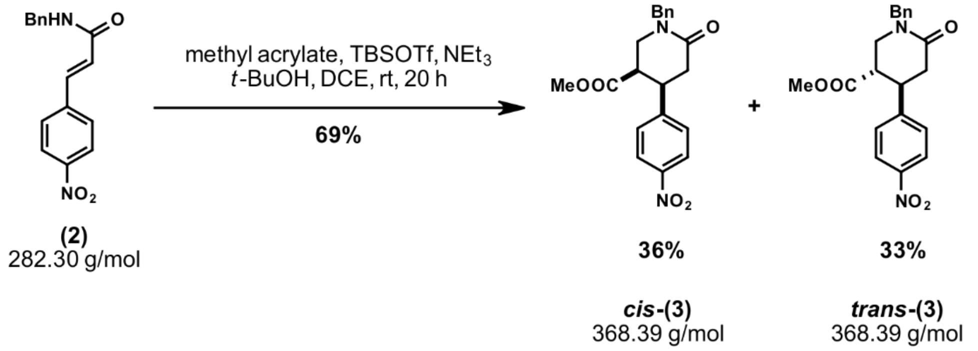

The piperidinone products ***cis*-(3)** and ***trans*-(3)** were prepared *via* a double Michael-addition in analogy to a literature procedure(Takasu et al., 2005). An oven-dried Schlenk flask was charged with amide **(2)** (565 mg, 2 mmol, 1 equiv.). The flask was closed with a septum and then evacuated and flushed with argon three times. Dry DCE (2.00 mL, 1.0 M relative to amide **(2)**), methyl acrylate (172 mg, 2 mmol, 1 equiv.) and dry NEt_3 (195 µL, 1.4 mmol, 0.7 equiv.) were added. TBSOTf (0.74 mL, 3.2 mmol, 1.6 equiv.) was added dropwise *via* syringe. Dry *t*-BuOH (0.05 mL, 0.5 mmol, 0.25 equiv.) was added subsequently to the suspension, in order to suppress the triple Michael adduct. The suspension was stirred at room temperature. In the course of the reaction the mixture became homogeneous. According to GC-MS analysis, the maximum of conversion was achieved after 20 h. The reaction mixture was quenched with 50 mL satd. aqu. $NaHCO_3$ and the aqu. phase was extracted with EtOAc (3x 30 mL) The combined organic phases were washed with brine (50 mL) and dried over $MgSO_4$. Volatiles were removed in *vacuo*, affording the crude material. Purification by flash column chromatography (silica gel/crude material = 100/1, EtOAc in LP 40% → 100%) afforded the desired products. In case of g-scale using the crude material without further purification was suitable, yielding only 6 % less of desired **trans-(3)** over two steps.

Yield 69% (506 mg, 1.37 mmol)

cis-(3): 36% (265 mg, 0.72 mmol)

trans-(3): 33% (241 mg, 0.65 mmol)

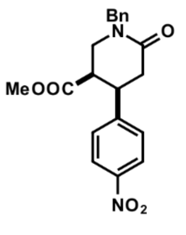

***cis*-(3)**

Appearance colorless crystals

Melting point 117.5 – 118.0 °C

TLC analysis R_f = 0.25 (LP/EtOAc = 1/2)

Sum formula $C_{20}H_{20}N_2O_5$

HRMS $[M+H]^+$: calculated: 369.1445 Da, found: 369.1469 Da, difference: 2.4 mDa

GC-MS 368 (33, M^+), 191 (17), 176 (23), 132 (13), 119 (25), 118 (13), 115 (10), 106 (14), 104 (11), 91 (100)

^{1}H-NMR (400 MHz, $CDCl_3$) δ = 2.97 (d, J = 5.7 Hz, 1H), 3.18 – 3.24 (m, 1H), 3.26 – 3.33 (m, 1), 3.44 (dd, J = 12.7, 4.9 Hz, 1H), 3.58 (s, 3H), 3.77 – 3.82 (m, 1H), 4.53 (d, J = 14.3 Hz, 1H), 4.82 (d, J = 14.3 Hz, 1H), 7.19 – 7.22 (m, 2H), 7.29 – 7.39 (m, 5H), 8.08 (d, J = 8.8 Hz, 2H) ppm.

^{13}C-NMR (101 MHz, $CDCl_3$) δ = 36.0 (t), 39.6 (d), 43.6 (d), 45.0 (t), 50.6 (t), 52.2 (q), 123.9 (d, 2C), 128.0 (d), 128.6 (d, 2C), 128.7 (d, 2C), 128.9 (d, 2C), 136.4 (s), 146.9 (s), 147.4 (s), 167.9 (s), 170.6 (s) ppm.

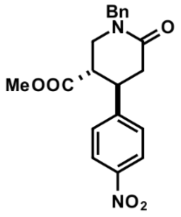

***trans*-(3)**

Appearance colorless crystals

Melting point 132.0 – 132.5 °C

TLC analysis R_f = 0.45 (LP/EtOAc = 1/2)

Sum formula $C_{20}H_{20}N_2O_5$

HRMS $[M+H]^+$: calculated: 369.1445 Da, found: 369.1461 Da, difference: 1.6 mDa

GC-MS 68 (23, M^+), 191 (12), 176 (17), 132 (10), 119 (14), 118 (10), 106 (12), 104 (10), 91 (100)

^{1}H-NMR (400 MHz, $CDCl_3$) δ = 2.65 (dd, J = 17.7, 10.7 Hz, 1H), 2.86 (dd, J = 17.7, 5.7 Hz, 1H), 3.05 (td, J = 9.9, 5.3 Hz, 1H), 3.42 (dd, J = 12.5, 5.3 Hz, 1H), 3.46 (s, 3H), 3.50 – 3.59 (m, 2H), 4.56 (d, J = 14.5 Hz, 1H), 4.76 (d, J = 14.5 Hz, 1H), 7.27 – 7.39 (m, 7H), 8.18 (d, J = 8.7 Hz, 2H) ppm.

^{13}C-NMR (101 MHz, $CDCl_3$) δ = 38.0 (t), 41.6 (d), 46.2 (d), 47.9 (t), 50.2 (t), 52.4 (q), 124.3 (d, 2C), 128.0 (d), 128.2 (d, 2C), 128.4 (d, 2C), 128.9 (d, 2C), 136.4 (s), 147.3 (s), 148.6 (s), 167.6 (s), 171.5 (s) ppm.

A II.1.4 Methyl (±)-*trans*-1-benzyl-4-(4-nitrophenyl)-6-oxopiperidine-3-carboxylate *trans*-(**3**)

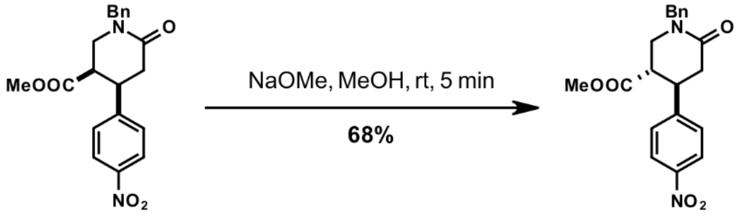

***cis*-(3)**
368.39 g/mol

***trans*-(3)**
368.39 g/mol

Sodium (34 mg, 1.50 mmol, 2.7 equiv.) was added to 4 mL dry MeOH under argon atmosphere. After full reaction of sodium to NaOMe, a solution of compound ***cis*-(3)** (200 mg, 0.54 mmol, 1 equiv.) in 5 mL dry MeOH was added dropwise *via* syringe. The reaction was stirred at room temperature. According to GC-MS analysis the equilibrium between the two diastereomers was reached after 5 min. The reaction mixture was quenched with 75 mL satd. aqu. NH_4Cl. After the addition of DCM (100 mL), H_2O (100 mL) was added to dissolve the precipitate. The phases were separated and the aqu. phase was extracted with DCM (3 x 50 mL). The combined organic phases were washed with satd. aqu. $NaHCO_3$ (100 mL) and brine (100 mL). Then the organic phase was dried over $MgSO_4$. Volatiles were removed in *vacuo* to afford the crude material. The product ***trans*-(3)** was obtained after purification *via* flash column chromatography (silica gel/crude material = 100/1, acetone in LP 20% → 50%).

Yield 68% (135 mg, 0.36 mmol)

Characterization of ***trans*-(3)** see previous section.

A II.1.5 (±)-*trans*-[1-Benzyl-4-(4-nitrophenyl)piperidin-3-yl]methanol *rac*-(**4**)

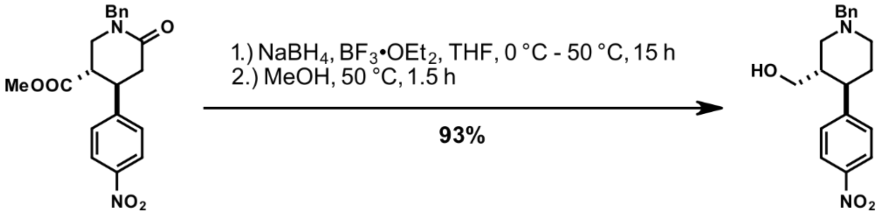

***trans*-(3)**
368.39 g/mol

***rac*-(4)**
326.40 g/mol

Alcohol ***rac*-(4)** was synthesized in analogy to a literature procedure (Norris et al., 2000). BF_3•OEt_2 (2.97 mL, 24.10 mmol, 4 equiv.) was added dropwise *via* syringe to a suspension of $NaBH_4$ (912 mg, 24.10 mmol, 4 equiv.) in dry THF (20.1 mL, 0.3 M relative to starting material) under argon at 0 °C. The mixture was stirred at 0 °C for 1 hour. Afterwards, a solution of ***trans*-(3)** (2.220 mg, 6.03 mmol, 1 equiv.) in dry THF (30.2 mL, 0.2 M relative to the starting material, final concentration: 0.1 M) was added *via* syringe. The resulting mixture was stirred at 50 °C

for 15 hours whereupon it was carefully quenched with 1 N NaOH (40 mL). The reaction was partitioned between EtOAc (400 mL) and 1 N NaOH (350 mL). Phases were separated and the aqu. phase was extracted with EtOAc (4 x 70 mL). The combined organic phases were washed with brine (250 mL). After drying over $MgSO_4$ and evaporation of the solvent, the borane complex of the desired product was obtained. MeOH (45 mL, 0.14 M relative to starting material) was added to the isolated material and the reaction was stirred at reflux temperature for 1.5 hours. Volatiles were removed in vacuo and the crude material was purified by vacuum filtration (silica gel/crude material = 15/1, washing step with DCM, eluent: DCM/MeOH = 10/1) affording an orange oil. After suspending the oil in DIPE followed by the removal of the solvent, orange crystals were obtained.

**Yield**	93% (1.83 g, 5.61 mmol)
**Appearance**	orange crystals
**Melting point**	104.0 – 105.0 °C
**TLC analysis**	R_f = 0.28 (DCM/MeOH = 10/1)
**Sum formula**	$C_{19}H_{22}N_2O_3$
**HRMS**	$[M+H]^+$: calculated: 327.1703 Da, found: 327.1729 Da, difference: 2.6 mDa
**^{1}H-NMR (400 MHz, $CDCl_3$)**	δ = 1.25 (s, 1H), 1.75 – 1.91 (m, 2H), 2.01 – 2.12 (m, 3H), 2.54 (td, *J* = 11.1, 4.5 Hz, 1H), 2.96 – 3.02 (m, 1H), 3.17 – 3.26 (m, 2H), 3.37 (dd, *J* = 10.8, 2.7 Hz, 1H), 3.56 (d, *J* = 13.1 Hz, 1H), 3.61 (d, *J* = 13.1 Hz, 1H), 7.26 – 7.37 (m, 5H), 7.39 (d, *J* = 8.8 Hz, 2H), 8.16 (d, *J* = 8.8 Hz, 2H) ppm.
**^{13}C-NMR (101 MHz, $CDCl_3$)**	δ = 34.2 (t), 44.0 (d), 45.0 (d), 53.8 (t), 57.2 (t), 63.6 (t), 63.8 (t), 124.0 (d, 2C), 127.3 (d), 128.4 (d, 2C), 128.5 (d, 2C), 129.3 (d, 2C), 138.2 (s), 146.8 (s), 152.7 (s) ppm.

A II.1.6 (3*S*,4*R*)-[1-Benzyl-4-(4-nitrophenyl)piperidin-3-yl]methanol (3*S*,4*R*)-(**4**)

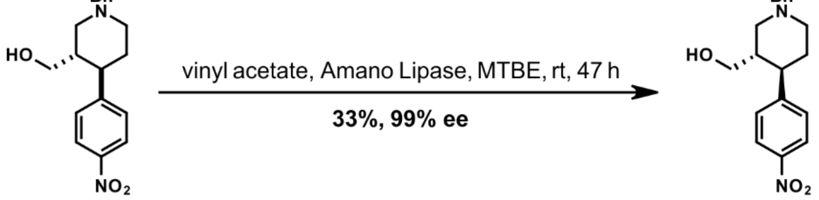

***rac*-(4)**
326.40 g/mol

(3*S*,4*R*)-(4)
326.40 g/mol

Vinyl acetate (14.95 mL, 161.46 mmol, 10 equiv.) and Amano Lipase PS (10.54 g, 200 wt.% immobilized on diatomite, product 708011 from Sigma-Aldrich) were added to a solution of ***rac*-(4)** (5.27 g, 16.15 mmol, 1 equiv.) in MTBE (808 mL, 0.02 M) at room temperature.

Conversion and changes in ee composition were monitored by chiral HPLC. The mixture was stirred at room temperature for 47 hours. Then the mixture was filtered through a pad of celite and DCM (1600 mL) was used for washing. Volatiles were removed in *vacuo*. Purification of the crude material by flash column chromatography (silica gel/crude material = 75/1, EtOAc in LP 0 --> 100) afforded pure alcohol **(3*S*,4*R*)-(4)**. Full conversion was necessary to reach a high ee.

Yield 33% (1.72 g, 5.27 mmol)

Characterization of **(3*S*,4*R*)-(4)** see previous section.

Enantiomeric excess (ee) 99%

Specific rotation $[\alpha]_D^{20}$ = -19.2 (c = 1.0, $CHCl_3$)

A II.1.7 (3R,4S)-[1-Benzyl-4-(4-nitrophenyl)piperidin-3-yl]methanol (*3R*,*4S*)-(**4**)

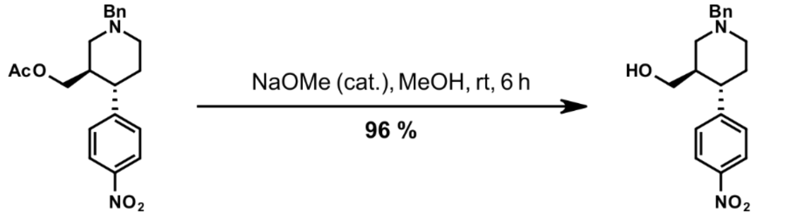

(3R,4S)-(5) 368.43 g/mol   **(3*R*,4*S*)-(4)** 326.40 g/mol

The deacetylation of acetate **(*3R,4S*)-(5)** was performed according to a modified literature procedure (Draskovits et al., 2018) in order to use the alcohol for test reactions. To a solution of acetate **(*3R,4S*)-(5)** (1.01 g, 2.74 mmol) in dry MeOH (40 mL, 0.07 M relative to **(*3R,4S*)-(5)**), a solution of NaOMe in dry MeOH ((30 % (w/v)) was added in catalytic amounts (approx. 0.1 mL) until a pH of 10 was reached (controlled *via* pH-paper). After 6 h (TLC control) the reaction was quenched with 50 mL aqu. solution of NH_4Cl and poured on additional 250 mL aqu. solution of NH_4Cl in order to neutralize the reaction mixture. H_2O (70 mL) was added to dissolve the formed precipitate. The aqu. layer was extracted with DCM (6 x 70 mL). The combined organic phases were washed with an aqu. solution of $NaHCO_3$ (1 x 100 mL) and brine (1 x 100 mL) and were dried over $MgSO_4$. Volatiles were removed in vacuo in order to afford the product **(*3R,4S*)-(4)** in high purity without any further purification.

Yield 96 % (859 mg, 9.96 mmol)

Characterization of **(3*R*,4*S*)-(4)** see previous section.

A II.1.8 (3*S*,4*R*)-3-[(Benzo[*d*][1,3]dioxol-5-yloxy)methyl]-1-benzyl-4-(4-nitrophenyl)piperidine (**6**)

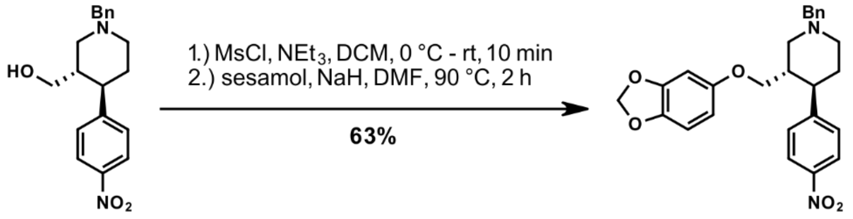

(3*S*,4*R*)-(4) 326.40 g/mol

(6) 446.50 g/mol

Ether **(6)** was synthesized in analogy to a literature procedure (Bower et al., 2007). Alcohol **(3*S*,4*R*)-(4)** (1.50 g, 4.60 mmol, 1 equiv.) was dissolved in dry DCM (23 mL, 0.2 M relative to alcohol **(3*S*,4*R*)-(4)**) and the solution was cooled to 0 °C under argon. Dry NEt_3 (0.92 mL, 6.63 mmol, 1.44 equiv.) and MsCl (0.51 mL, 6.63 mmol, 1.44 equiv.) were added *via* syringe. The mixture was stirred at room temperature for 10 min and afterwards it was quenched with H_2O (50 mL). The quenched mixture was poured on H_2O (50 mL) and satd. aqu. $NaHCO_3$ (100 mL). The aqu. phase was extracted with DCM (8 x 50 mL). The combined organic phase was washed with brine (80 mL) and dried over $MgSO_4$. After evaporation of the solvent the crude mesylate was obtained. NaH (368 mg, 60% dispersion in mineral oil, 9.20 mmol, 2 equiv.) was added to a solution of sesamol (1.27 g, 9.20 mmol, 2 equiv.) in dry DMF (38 mL, 0.24 M relative to sesamol) at 0 °C under argon. The mixture was stirred at room temperature for 20 minutes. A solution of the mesylate in dry DMF (19 mL, 0.24 M relative to the mesylate) was added to the phenolate solution *via* syringe. The reaction mixture was stirred at 90 °C for 2 hours. The reaction was diluted with EtOAc (200 mL) and washed with H_2O (100 mL) and 1 N NaOH (3 x 100 mL). The combined organic phases were washed with brine (100 mL) and dried over $MgSO_4$. The solvent was removed in *vacuo* and the crude product was purified by flash column chromatography (silica gel/crude material = 100/1, EtOAc in LP 0% → 40%) yielding the desired product **(6)**.

**Yield**	63% (1.29 g, 2.89 mmol)
**Appearance**	orange oil
**TLC analysis**	R_f = 0.65 (LP/EtOAc = 1/2)
**Sum formula**	$C_{26}H_{26}N_2O_5$
**HRMS**	$[M+H]^+$: calculated: 447.1915 Da, found: 447.1946 Da, difference: 3.1 mDa
**^{1}H-NMR (400 MHz, $CDCl_3$)**	δ = 1.78 – 1.97 (m, 2H), 2.07 – 2.21 (m, 2H), 2.23 – 2.35 (m, 1H), 2.70 (td, *J* = 11.6, 4.3 Hz, 1H), 2.99 – 3.07 (m, 1H), 3.18 – 3.26 (m, 1H), 3.43 (dd, *J* = 9.4, 6.1 Hz, 1H), 3.53 (dd, *J* = 9.4, 3.0 Hz, 1H), 3.57 (d, *J* = 13.1 Hz, 1H), 3.65 (d, *J* = 13.1 Hz, 1H), 5.88 (s, 2H),

6.08 (dd, *J* = 8.5, 2.5 Hz, 1H), 6.29 (d, *J* = 2.5 Hz, 1H), 6.61 (d, *J* = 8.5 Hz, 1H), 7.27 – 7.40 (m, 7H), 8.14 (d, *J* = 8.8 Hz, 2H) ppm.

**^{13}C-NMR (101 MHz, CDCl$_3$)**	δ = 34.0 (t), 42.1 (d), 44.9 (d), 53.7 (t), 57.3 (t), 63.4 (t), 69.4 (t), 98.0 (d), 101.2 (t), 105.5 (d), 108.0 (d), 124.0 (d, 2C), 127.3 (d), 128.4 (d, 2C), 128.5 (d, 2C), 129.3 (d, 2C), 138.1 (s), 141.8 (s), 146.8 (s), 148.3 (s), 152.3 (s), 154.3 (s) ppm.
**Specific rotation**	$[\alpha]_D^{20}$ = -74.2 (c = 1.0, CHCl$_3$)

A II.1.9 4-{(3*S*,4*R*)-3-[(Benzo[*d*][1,3]dioxol-5-yloxy)methyl]piperidin-4-yl}aniline (**7**)

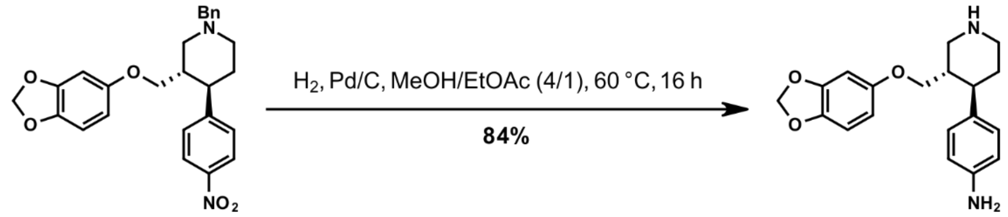

(6)
446.50 g/mol

(7)
326.40 g/mol

10% Pd/C (19 mg, 10 mol%) was added to a solution of the starting material **(6)** (78 mg, 0.175 mmol) in MeOH/EtOAc (1.75 mL, 4/1). The mixture was stirred under an atmosphere of H_2 at 60 °C for 16 hours. The mixture was filtered through a pad of celite (MeOH was used for washing). Volatiles were removed in *vacuo* and the crude material was purified by flash column chromatography (silica gel/crude material = 100/1, CHCl$_3$/MeOH/NH$_4$OH = 80/20/1) affording the desired aniline **(7)**.

**Yield**	84% (48 mg, 0.147 mmol)
**Appearance**	pale yellow oil
**TLC analysis**	R_f = 0.25 (CHCl$_3$/MeOH/NH$_4$OH = 80/20/1)
**Sum formula**	$C_{19}H_{22}N_2O_3$
**HRMS**	$[M+H]^+$: calculated: 327.1703 Da, found: 327.1720 Da, difference: 1.7 mDa
**^{1}H-NMR (400 MHz, CDCl$_3$)**	δ = 1.65 – 1.83 (m, 2H), 2.00 – 2.10 (m, 1H), 2.45 (td, *J* = 11.6, 4.2 Hz, 1), 2.64 (t, *J* = 11.6 Hz, 1H), 2.73 (td, *J* = 12.0, 2.9 Hz, 1H), 3.18 (d, *J* = 12.3 Hz, 1H), 3.40 – 3.47 (m, 2H), 3.61 (dd, *J* = 9.4, 3.0 Hz, 1H), 5.86 (s, 2H), 6.13 (dd, *J* = 8.5, 2.5 Hz, 1H), 6.34 (d, *J* = 2.5 Hz, 1H), 6.59 – 6.64 (m, 3H), 6.98 (d, *J* = 8.3 Hz, 2H) ppm.

**^{13}C-NMR (101 MHz, $CDCl_3$)**	δ = 35.4 (t), 42.9 (d), 44.5 (d), 47.2 (t), 50.5 (t), 69.8 (t), 98.1 (d), 101.1 (t), 105.7 (d), 107.9 (d), 115.5 (d, 2C), 128.3 (d, 2C), 134.3 (s), 141.5 (s), 144.9 (s), 148.2 (s), 154.6 (s) ppm.
**Specific rotation**	$[\alpha]_D^{20}$ = -72.1 (c = 1.0, $CHCl_3$)

A II.1.10 (3*S*,4*R*)-3-[(Benzo[*d*][1,3]dioxol-5-yloxy)methyl]-4-[4-((*E*)-phenyldiazenyl)phenyl]piperidine (**9**)

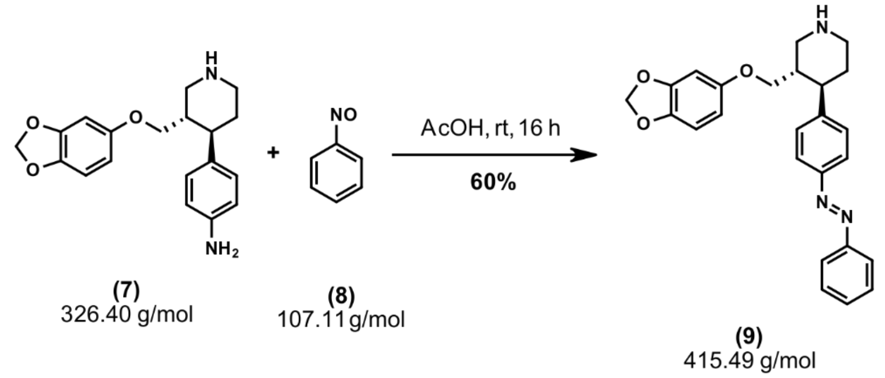

Nitrosobenzene **(8)** (31.5 mg, 0.294 mmol, 2 equiv.) was added to a solution of aniline **(7)** (48 mg, 0.147 mmol, 1 equiv.) in AcOH (1.47 mL, 0.1 M) and the solution was stirred under argon at room temperature for 16 hours. The solution was basified with 1 N NaOH and the mixture was extracted with $CHCl_3$ (3 x 5 mL). The combined organic phases were washed with H_2O (10 mL) and dried over $MgSO_4$. After evaporation of the solvent the crude material was purified by flash column chromatography (silica gel/crude material = 100/1, $CHCl_3$/MeOH/NH_4OH = 100/10/1) affording the desired azo compound **(9)**.

**Yield**	60% (36.5 mg, 0.088 mmol)
**Appearance**	orange oil
**TLC analysis**	R_f = 0.55 ($CHCl_3$/MeOH/NH_4OH = 80/20/1)
**Sum formula**	$C_{19}H_{22}N_2O_3$
**HRMS**	$[M+H]^+$: calculated: 416.1969 Da, found: 416.1987 Da, difference: 1.8 mDa
**^{1}H-NMR (400 MHz, $CDCl_3$)**	δ = 1.78 – 1.94 (m, 2H), 2.15 – 2.26 (m, 1H), 2.68 – 2.86 (m, 3H), 3.26 (d, *J* = 12.2 Hz, 1H), 3.45 – 3.52 (m, 2H), 3.62 (dd, *J* = 9.5, 3.0 Hz, 1H), 5.87 (s, 2H), 6.13 (dd, *J* = 8.5, 2.5 Hz, 1H), 6.35 (d, *J* = 2.5 Hz, 1H), 6.61 (d, *J* = 8.5 Hz, 1H), 7.37 (d, *J* = 8.4 Hz, 2H), 7.44 – 7.48 (m, 1H), 7.48 – 7.54 (m, 2H), 7.86 (d, *J* = 8.4 Hz, 2H), 7.88 – 7.91 (m, 2H) ppm.

^{13}C-NMR (101 MHz, $CDCl_3$) δ = 34.8 (t), 42.6 (d), 45.2 (d), 46.9 (t), 50.2 (t), 69.5 (t), 98.1 (d), 101.2 (t), 105.6 (d), 107.9 (d), 122.9 (d, 2C), 123.3 (d, 2C), 128.3 (d, 2C), 129.2 (d, 2C), 131.0 (d), 141.7 (s), 147.6 (s), 148.2 (s), 151.6 (s), 152.8 (s), 154.4 (s) ppm.

Specific rotation $[\alpha]_D^{20}$ = -109.9 (c = 0.5, $CHCl_3$)

A II.1.11 Determination of absolute configuration

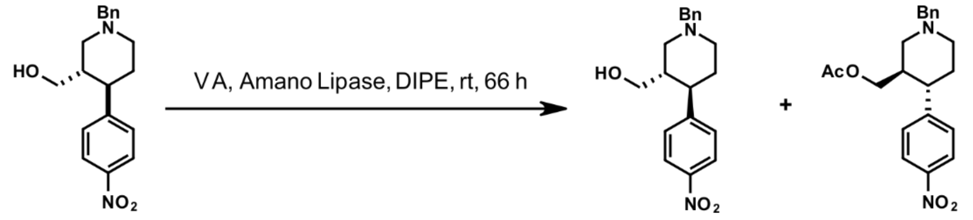

***rac*-(4)** 326.40 g/mol

(3*S*,4*R*)-(4) 326.40 g/mol — 43%, 56% ee

(3*R*,4*S*)-(5) 368.43 g/mol — 55%, 89% ee

Vinyl acetate (264 mg, 3.06 mmol, 10 equiv.) and Amano Lipase PS (100 mg, immobilized on diatomite, product 708011 from Sigma-Aldrich) were added to a solution of ***rac*-(4)** (100 mg, 0.31 mmol, 1 equiv.) in DIPE (15.3 mL, 0.02 M) at room temperature. Conversion and changes in ee composition were monitored by chiral HPLC. The mixture was stirred at room temperature for 66 hours reaching 55% conversion. Then the mixture was filtered through pad of celite and DIPE was used for washing. Volatiles were removed in *vacuo*. Purification of the crude material by flash column chromatography (silica gel/crude material = 100/1, EtOAc in LP 20 --> 100) afforded 43 mg of unreacted alcohol with 56% ee and 62 mg of acetate with 89% ee.

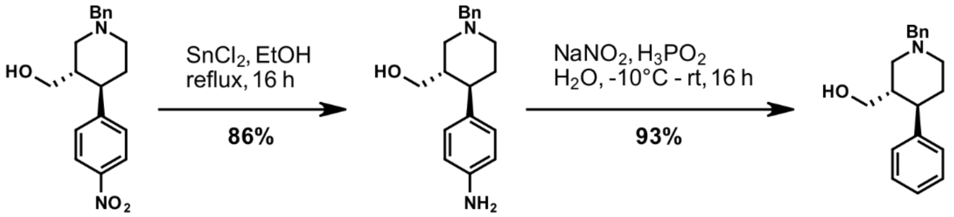

56% ee

$SnCl_2$ (100 mg, 0.53 mmol, 4 equiv.) was added to a solution of the obtained alcohol (43 mg, 0.13 mmol, 1 equiv., 56% ee) in EtOH (1.32 mL) and the mixture was stirred at reflux temperature for 16 hours. Volatiles were removed in vacuo. The residue was partitioned between EtOAc (2 mL) and satd. aqu. $NaHCO_3$ (2 mL). The mixture was filtered through a pad of celite and EtOAc was used for washing. Phases were separated. The organic phase was

washed with brine and dried over $MgSO_4$. After evaporation of the solvent 33.5 mg (86%) of the aniline analog were obtained. The material was dissolved in 50% H_3PO_2 (1.11 mL) and H_2O (0.37 mL) and the solution was cooled to -10 °C. A solution of $NaNO_2$ (8.4 mg, 0.12 mmol, 1 equiv.) in H_2O (0.186 mL) was added to this solution. The reaction was stirred for 16 hours and subsequently partitioned between satd. aqu. $NaHCO_3$ (50 mL) and EtOAc (25 mL). Phases were separated and the aqu. phase was extracted with EtOAc (25 mL). The combined organic phases were washed with brine and dried over $MgSO_4$. After evaporation of the solvent 29 mg (93%) of the product were obtained as a yellow oil. The specific optical rotation was determined ($[\alpha]_D^{23}$ = -8.2, c = 1.0, $CHCl_3$, 56% ee) and compared with the literature(Brandau et al., 2006) value for the compound with an absolute configuration of (3*R*,4*S*) ($[\alpha]_D^{23}$ = +15.1, c = 1.0, $CHCl_3$, 90% ee). As the synthesized material had the opposite optical rotation it was concluded that the Amano Lipase preferably acetylates the alcohol with (3*R*,4*S*) configuration and, hence, the remaining alcohol can be obtained in the desired (3*S*,4*R*) configuration.

**^{1}H-NMR (400 MHz, $CDCl_3$)**	δ = 1.76 – 1.83 (m, 1H), 1.86 – 1.97 (m, 1H), 2.00 – 2.15 (m, 3H), 2.35 (td, *J* = 11.5, 4.1 Hz, 1H), 2.98 – 3.04 (m, 1H), 3.20 – 3.30 (m, 2H), 3.39 (dd, *J* = 10.9, 3.2 Hz, 1H), 3.59 (d, *J* = 13.1 Hz, 1H), 3.67 (d, *J* = 13.1 Hz, 1H), 7.19 – 7.40 (m, 10H) ppm.
**Comment**	Spectral data are in accordance with the literature.(Brandau et al., 2006; Kim et al., 2016)

A III Kinetic Data

The rough experimental half-life time of the (*Z*)-isomer of compound **9** was determined on a UV-1800 UV/Vis spectrophotometer from Shimadzu at 23 °C. A 50 µM sample of the compound in dry DMSO was irradiated with 365 nm for 5 seconds using the respective OmniCure® LED head at 100% power. Once the highest possible (*Z*)-isomer content was reached, the absorption at 331 nm, the (*E*)-isomer's absorption maximum l_{max}, was regularly measured over the course of 12 hours. This data was used for the half-life time determination of the thermal (*Z*)/(*E*) isomerization.
A mathematical fitting function was used to linearize the obtained absorption data over time according to Equation S1.

$$A_{linearized} = \ln\left(A_{max} - A_t\right)$$

Equation S1. Linear fit used for absorption A.

The linearized absorption value gained by equation S1 was plotted over time and a linear fit function was applied. The obtained fit function gave the rate constant k as its slope value. Next, the half-life time was calculated according to Equation S2. The resulting graph is shown in Graph S1, the resulting half-life time was determined to be 6.5 days ($k = 1.24 * 10^{-6} s^{-1}$).

$$\tau_{1/2} = \frac{\ln(2)}{k}$$

Equation S2. Calculation of half-life $\tau_{1/2}$ time from rate constant k.

This method of thermal half-life time determination was sufficiently precise for the investigated compound, as the observed half-life time is in the range of days. This method has been shown (Singer et al, 2023) to yield similar results as compiling an Eyring plot for half-life time determination without the compound relaxing back to nearly 100% (*E*)-isomer.

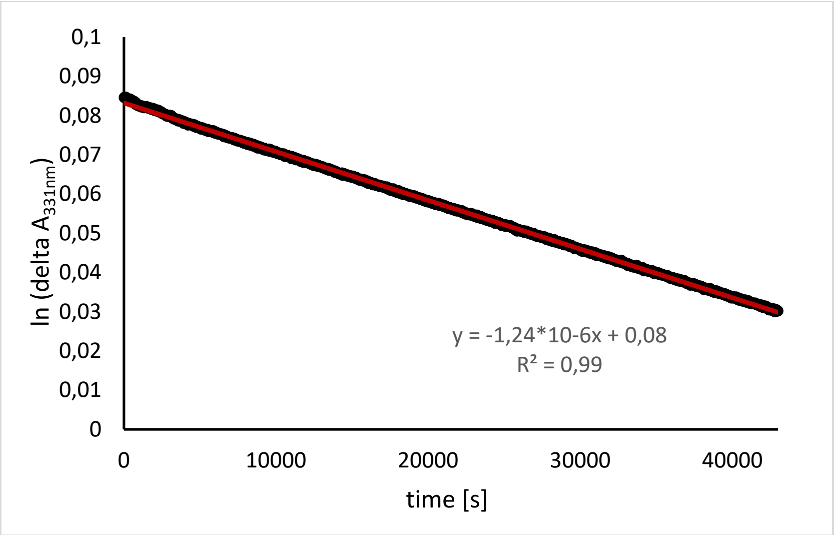

Graph S1. Experimental kinetic data for the thermal (*Z*)/(*E*) isomerization of compound **9**.

A IV Molecular Modeling

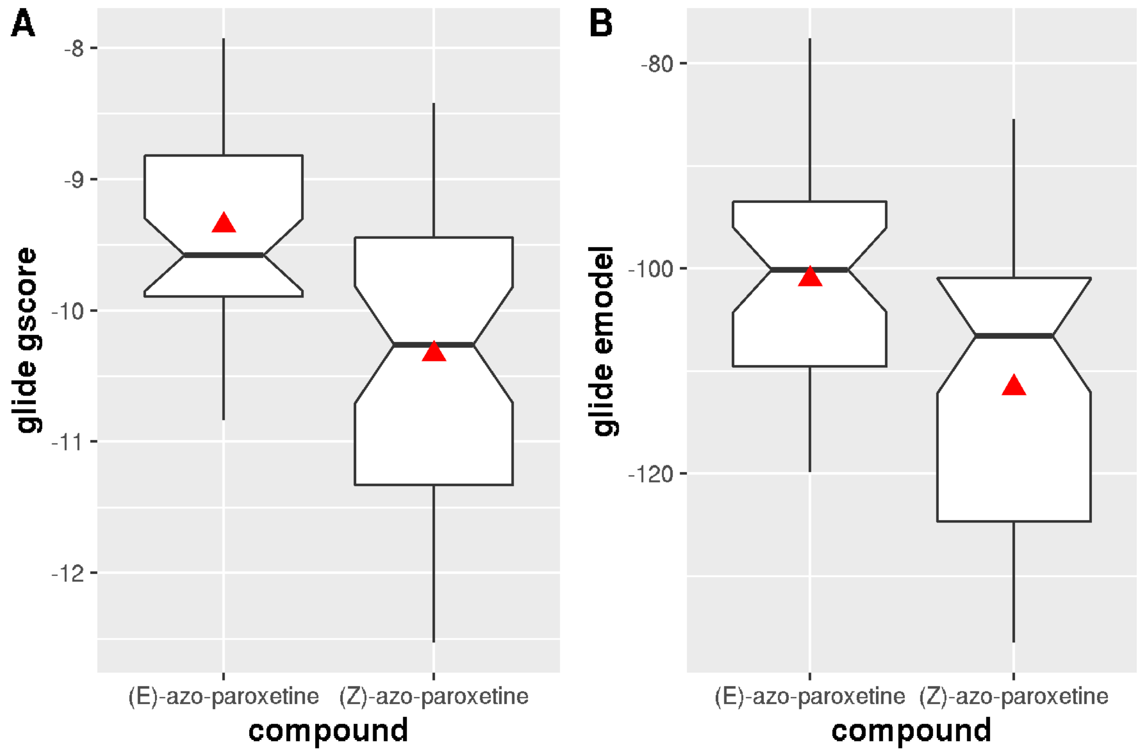

Figure S1: A: Distribution of glide gscore among all docking poses**.** The boxplots show the median as bold line and the mean as red triangle. The upper hinge represents the 75th percentile and the lower hinge represents the 25th percentile. The whiskers represent the 1.5 inter quartile range. The notches represent 95% confidence interval around the median. **B:** Distribution of glide emodel score among all docking poses.

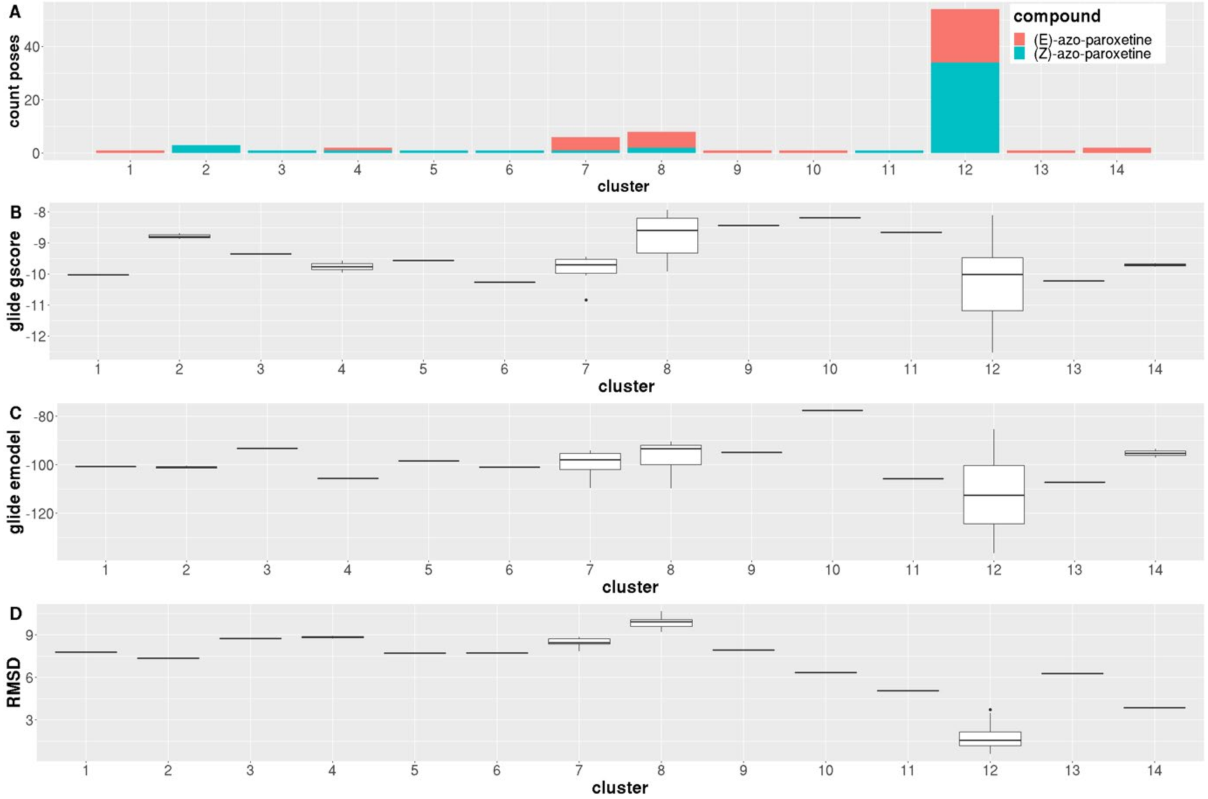

Figure S2: A: Distribution of poses per cluster. The boxplots show the median as bold line**.** The upper hinge represents the 75th percentile and the lower hinge represents the 25th percentile. The whiskers represent the 1.5 inter quartile range. **B:** Distribution of glide gscore per cluster. **C**: Distribution of glide emodel score per cluster. **D:** Distribution of RMSD in Å per cluster.

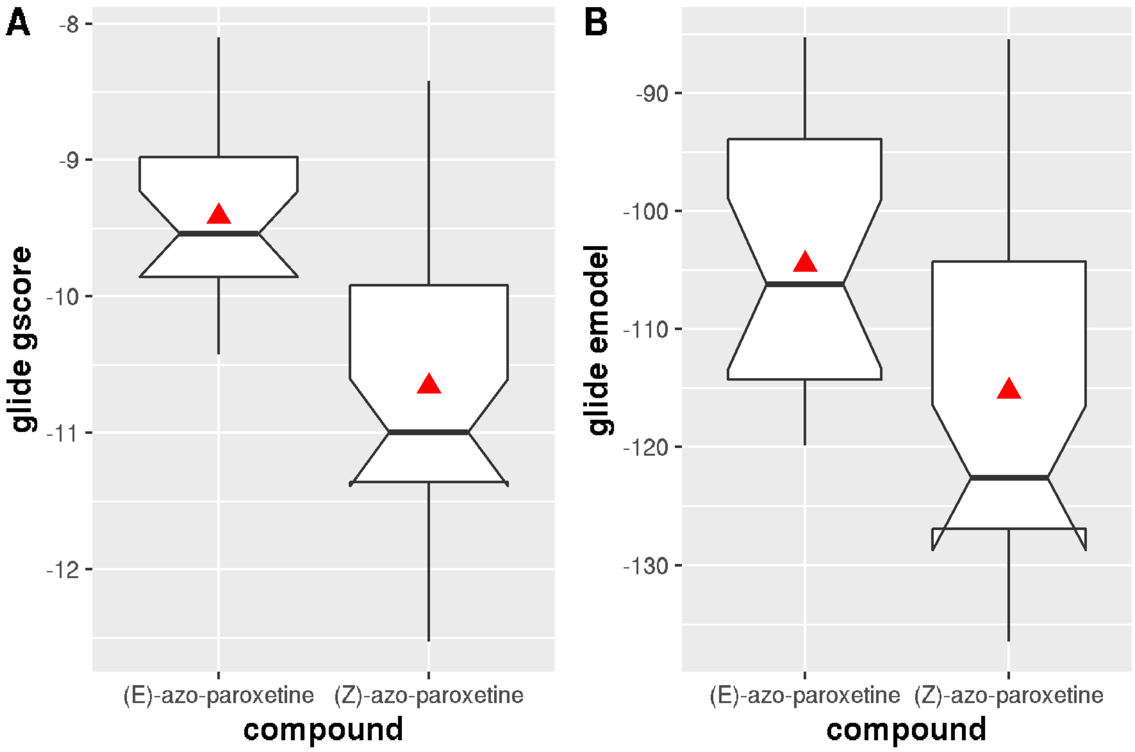

Figure S3: A. Distribution of glide gscores among poses of the most populated cluster (12). The boxplots show the median as bold line and the mean as red triangle. The upper hinge represents the 75th percentile and the lower hinge represents the 25th percentile. The whiskers represent the 1.5 inter quartile range. The notches represent 95% confidence interval around the median. **B.** Distribution of glide emodel score among poses of the most populated cluster (12).

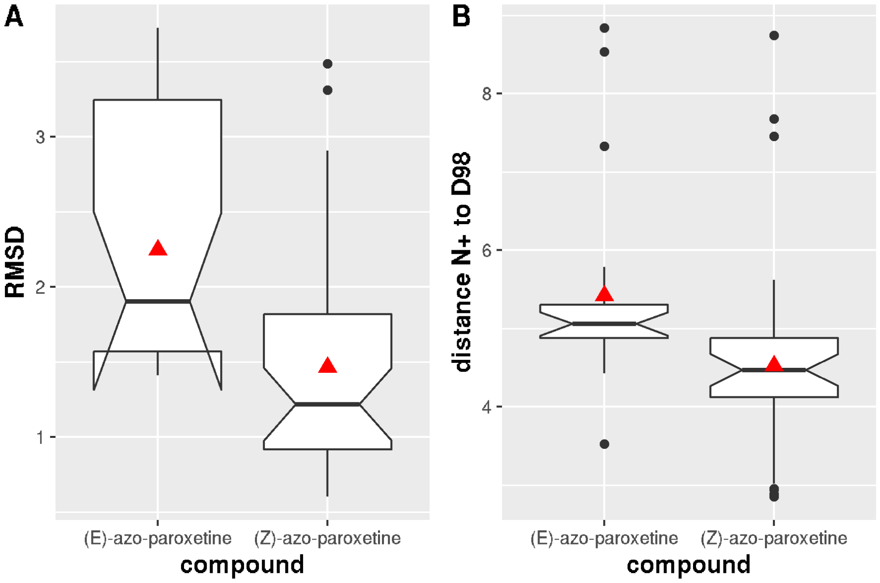

Figure S4: A: Distribution of RMSD in Å of all poses in the most populated cluster (12). The boxplots show the median as bold line and the mean as red triangle. The upper hinge represents the 75th percentile and the lower hinge represents the 25th percentile. The

whiskers represent the 1.5 inter quartile range. The notches represent 95% confidence interval around the median. **B:** Distribution of distances (in Å) of the cationic nitrogen to the charged sidechain oxygen of D98 of all poses in the most populated cluster (12).

Table 1: MM-GBSA calculations (dG bind in kcal/mol), glide gscores, glide emodel scores, RMSD in Å from paroxetine scaffold and distance between the cationic nitrogen of the ligand and the charged sidechain oxygen of D98 of selected poses of the most populated cluster (12). Poses that showed the best dG were selected as most promising and are depicted in bold.

**pose**	**emodel**	**gscore**	**RMSD** (Å)	**Distance** (Å)	**distance** (after Prime optimization)	**dG bind** (kcal/mol)
Z1	-136.5	-12.5	1.0	3.0	2.9	-139.77
Z2	-130.6	-11.6	0.9	4.2	4.2	-134.77
**Z3**	**-128.9**	**-11.5**	**0.9**	**4.4**	**4.3**	**-141.67**
Z4	-128.8	-11.8	1.2	4.2	4.1	-132.19
Z5	-127.4	-11.4	0.9	4.3	4.4	-142.39
Z6	-124.9	-11.2	0.9	4.2	4.2	-134.6
E1	-118.7	-10.2	2.1	4.4	4.8	-122.97
**E2**	**-110.4**	**-9.7**	**1.5**	**5.0**	**5.1**	**-125.79**
E3	-109.6	-9.5	1.5	5.1	5.2	-115.34
E4	102.8	-10.1	1.4	4.7	4.8	-110.02

Reference list:

Bower JF, Riis-Johannessen T, Szeto P, Whitehead AJ and Gallagher T (2007) Stereospecific construction of substituted piperidines. Synthesis of (-)-paroxetine and (+)-laccarin. *Chemical Communications*: 728 - 730.10.1039/b617260a

Brandau S, Landa A, Franzen J, Marigo M and Jorgensen KA (2006) Organocatalytic conjugate addition of malonates to α,β-unsaturated aldehydes: asymmetric formal synthesis of (-)-paroxetine, chiral lactams, and lactones. *Angew Chem, Int Ed* **45**: 4305-4309.10.1002/anie.200601025

Draskovits M, Stanetty C, Baxendale IR and Mihovilovic MD (2018) Indium- and Zinc-Mediated Acyloxyallylation of Protected and Unprotected Aldotetroses—Revealing a Pronounced Diastereodivergence and a Fundamental Difference in the Performance of the Mediating Metal. *The Journal of Organic Chemistry* **83**: 2647-2659.10.1021/acs.joc.7b03063

Kim JH, Shyam PK, Kim MJ, Lee H-J, Lee JT and Jang H-Y (2016) Enantioselective synthesis and antioxidant activity of 3,4,5-substituted piperidine derivatives. *Bioorg Med Chem Lett* **26**: 3119-3121.10.1016/j.bmcl.2016.04.092

Norris T, Braish TF, Butters M, DeVries KM, Hawkins JM, Massett SS, Rose PR, Santafianos D and Sklavounos C (2000) Synthesis of trovafloxacin using various (1α,5α,6α)-3-azabicyclo[3.1.0]hexane derivatives. *Perkin 1*: 1615-1622.

Pardin C, Pelletier JN, Lubell WD and Keillor JW (2008) Cinnamoyl inhibitors of tissue transglutaminase. *Journal of Organic Chemistry* **73**: 5766 - 5775.10.1021/jo8004843

Singer NK, Schlögl K, Zobel JP, Mihovilovic MD, González L (2023) Singlet and Triplet Pathways Determine the Thermal Z/E Isomerization of an Arylazopyrazole-Based Photoswitch. *The Journal of Physical Chemical Letters* **14**, 8956–8961 10.1021/acs.jpclett.3c01785

Takasu K, Nishida N, Tomimura A and Ihara M (2005) Convenient synthesis of substituted piperidinones from alpha,beta- unsaturated amides: Formal synthesis of deplancheine, tacamonine, and paroxetine. *Journal of Organic Chemistry* **70**: 3957 - 3962.10.1021/jo050261x

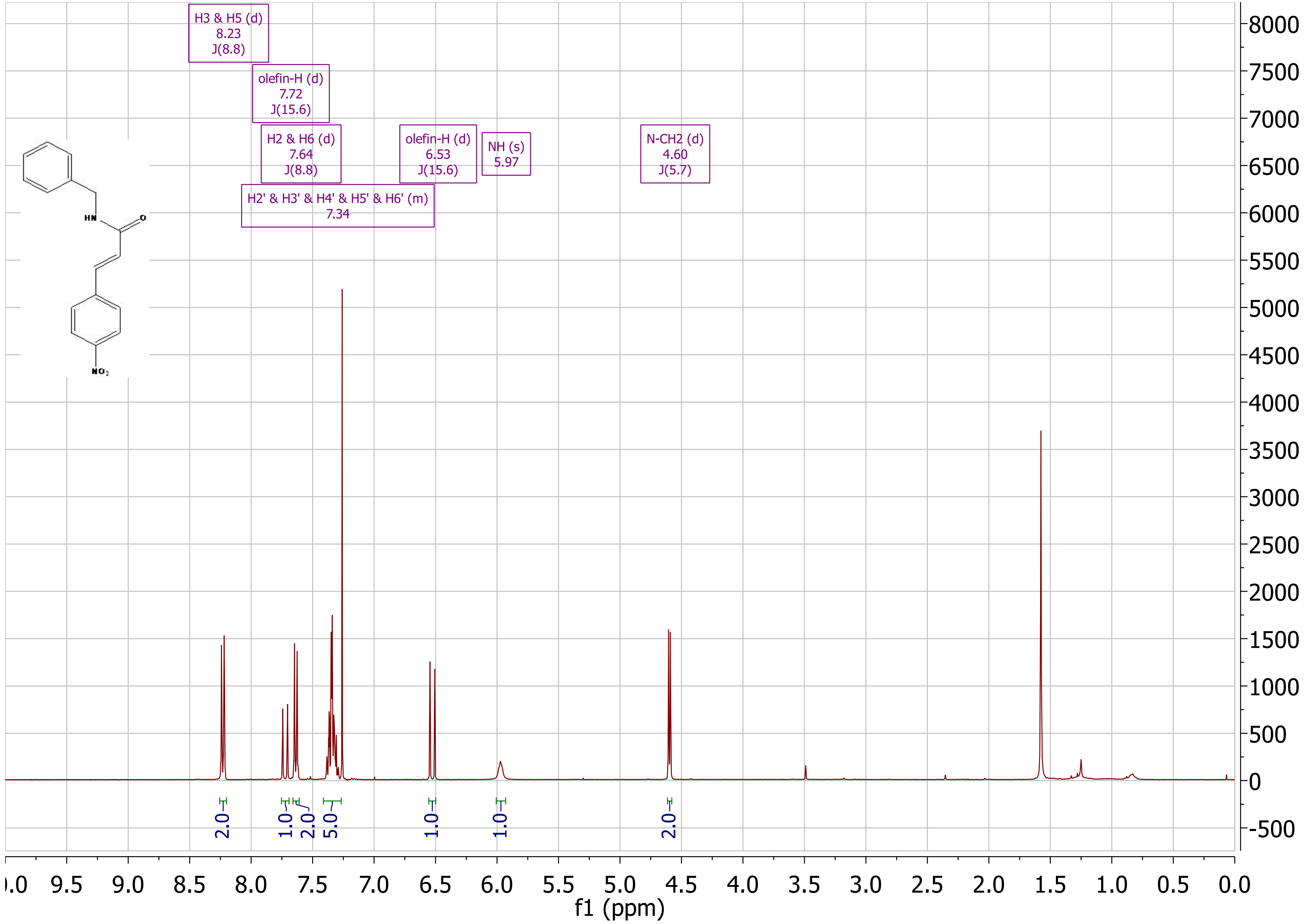

H3 & H5 (d)
8.23
J(8.8)
olefin-H (d)
7.72
J(15.6)
H2 & H6 (d)
7.64
J(8.8)
olefin-H (d)
6.53
J(15.6)
NH (s)
5.97
N-CH2 (d)
4.60
J(5.7)
H2' & H3' & H4' & H5' & H6' (m)
7.34
HN
O
NO2
2.0
1.0
2.0
5.0
1.0
1.0
2.0
f1 (ppm)

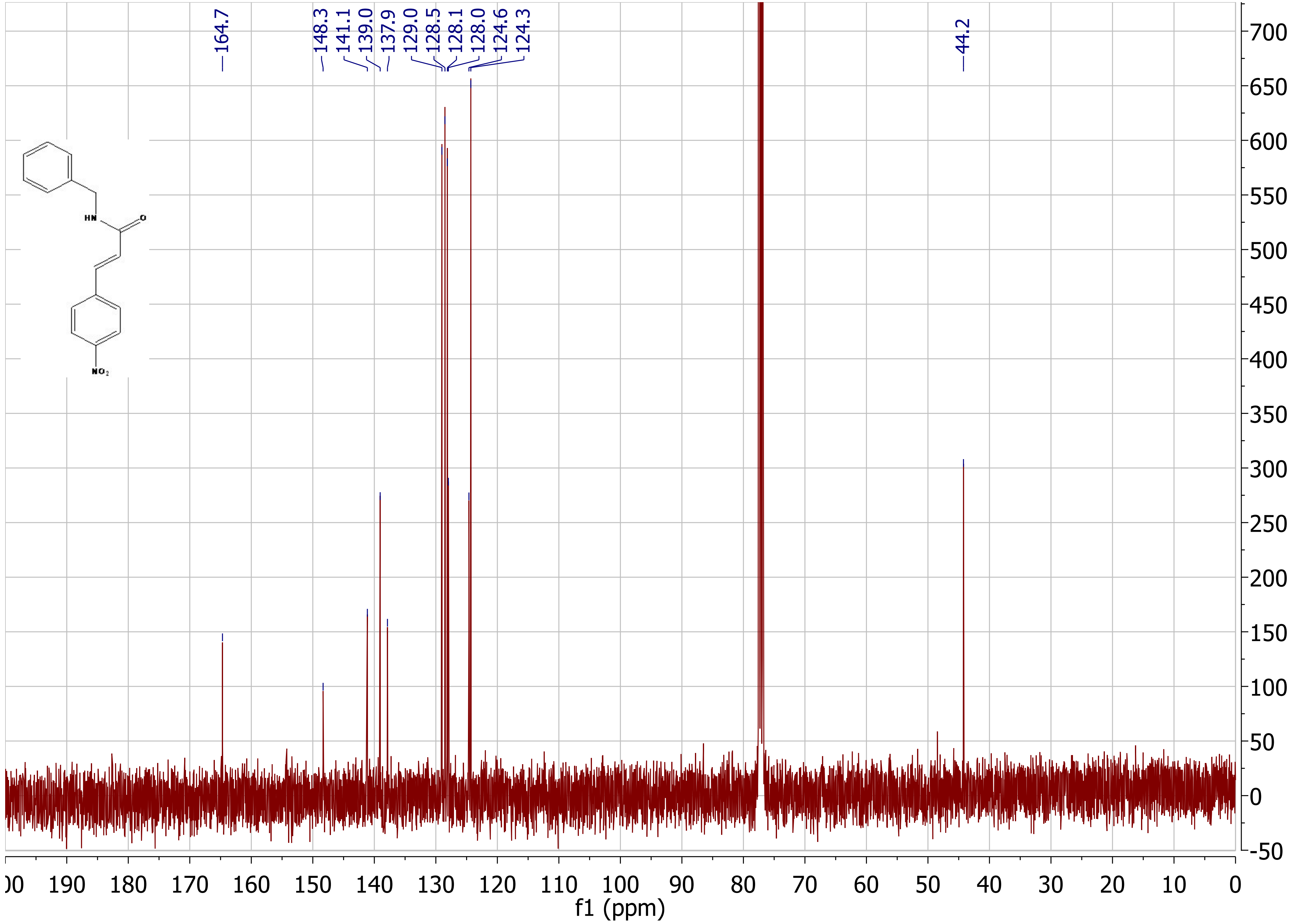
164.7
148.3
141.1
139.0
137.9
129.0
128.5
128.1
128.0
124.6
124.3
44.2
HN
O
NO2
f1 (ppm)

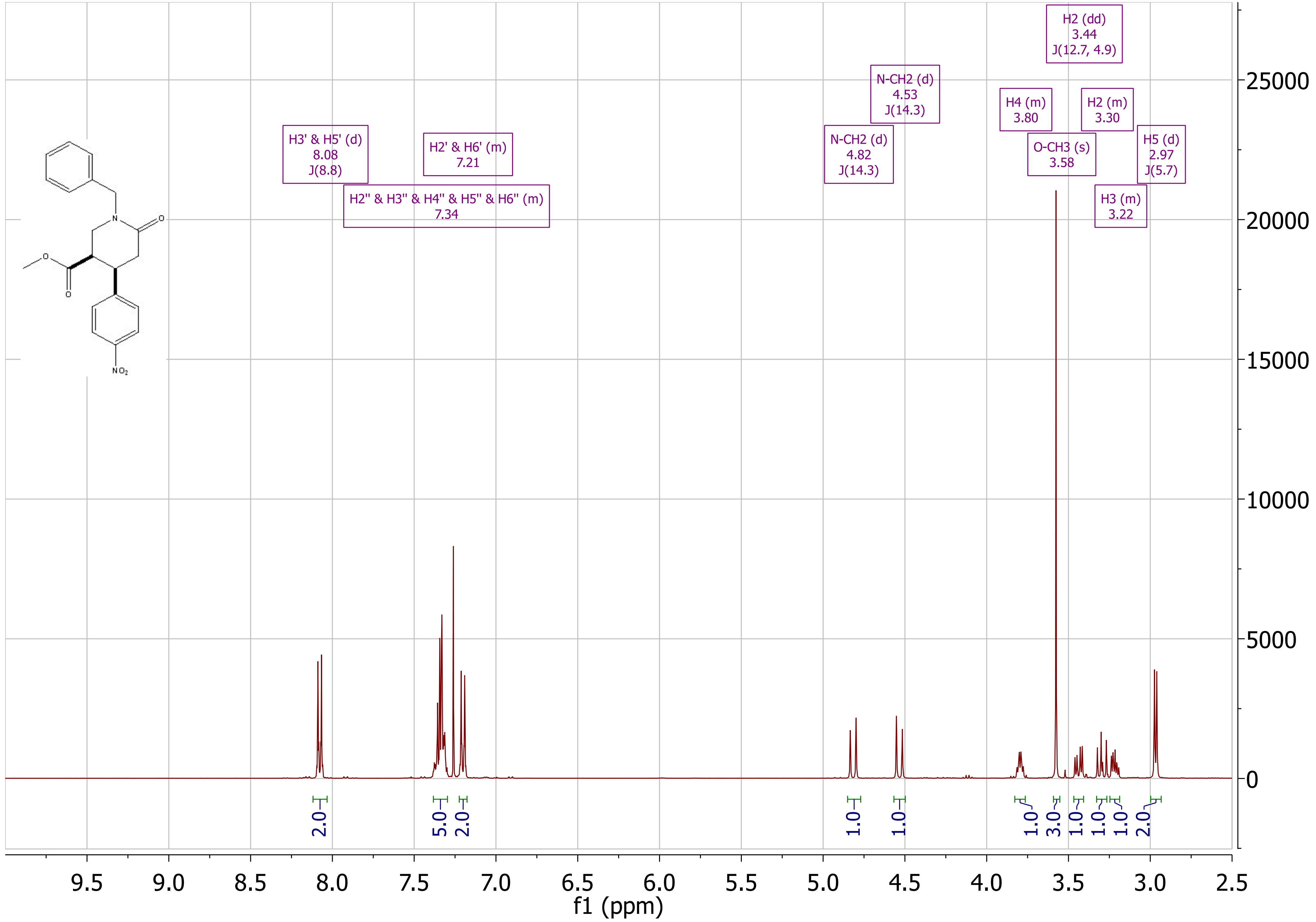

H2 (dd)
3.44
J(12.7, 4.9)
N-CH2 (d)
4.53
J(14.3)
H4 (m)
3.80
H2 (m)
3.30
H3' & H5' (d)
8.08
J(8.8)
H2' & H6' (m)
7.21
N-CH2 (d)
4.82
J(14.3)
O-CH3 (s)
3.58
H5 (d)
2.97
J(5.7)
H2'' & H3'' & H4'' & H5'' & H6'' (m)
7.34
H3 (m)
3.22
2.0
5.0
2.0
1.0
1.0
1.0
3.0
1.0
1.0
1.0
2.0
25000
20000
15000
10000
5000
0
9.5
9.0
8.5
8.0
7.5
7.0
6.5
6.0
5.5
5.0
4.5
4.0
3.5
3.0
2.5
f1 (ppm)
NO2

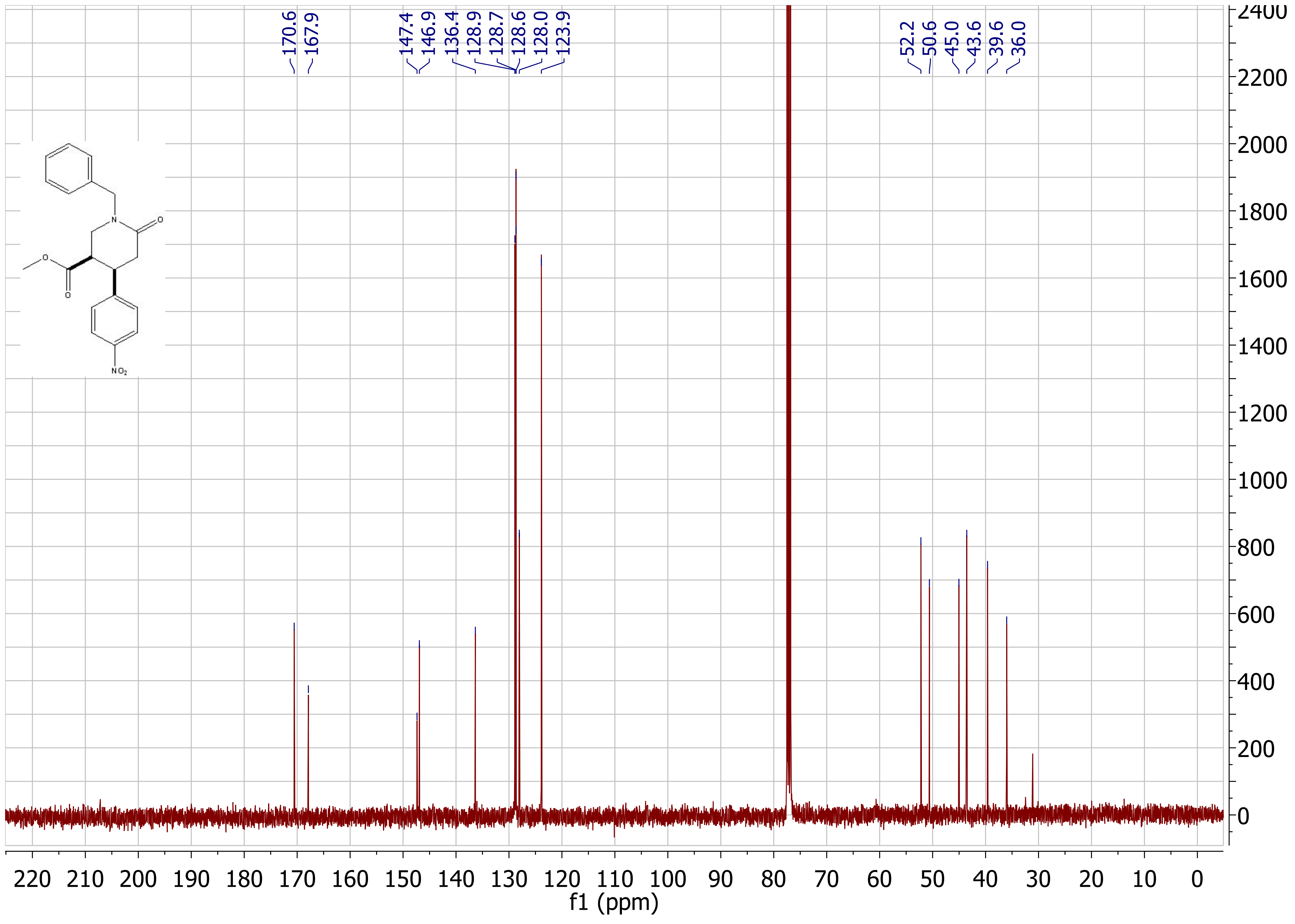
170.6
167.9
147.4
146.9
136.4
128.9
128.7
128.6
128.0
123.9
52.2
50.6
45.0
43.6
39.6
36.0
N
O
O
O
NO2
220 210 200 190 180 170 160 150 140 130 120 110 100 90 80 70 60 50 40 30 20 10 0
f1 (ppm)
2400
2200
2000
1800
1600
1400
1200
1000
800
600
400
200
0

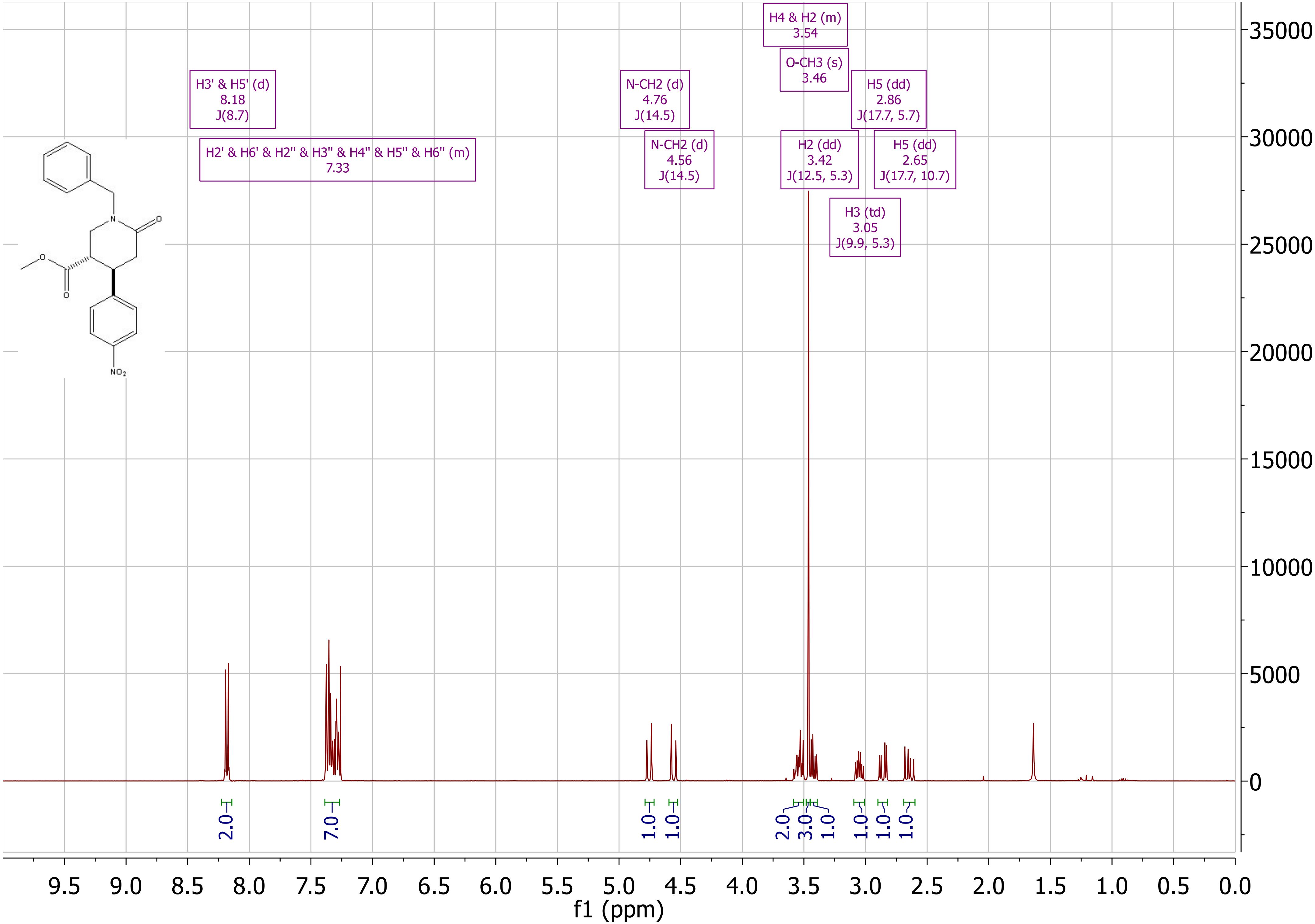
H4 & H2 (m)
3.54
O-CH3 (s)
3.46
H3' & H5' (d)
8.18
J(8.7)
N-CH2 (d)
4.76
J(14.5)
H5 (dd)
2.86
J(17.7, 5.7)
H2' & H6' & H2'' & H3'' & H4'' & H5'' & H6'' (m)
7.33
N-CH2 (d)
4.56
J(14.5)
H2 (dd)
3.42
J(12.5, 5.3)
H5 (dd)
2.65
J(17.7, 10.7)
H3 (td)
3.05
J(9.9, 5.3)
NO2
2.0
7.0
1.0
1.0
2.0
3.0
1.0
1.0
1.0
1.0
f1 (ppm)

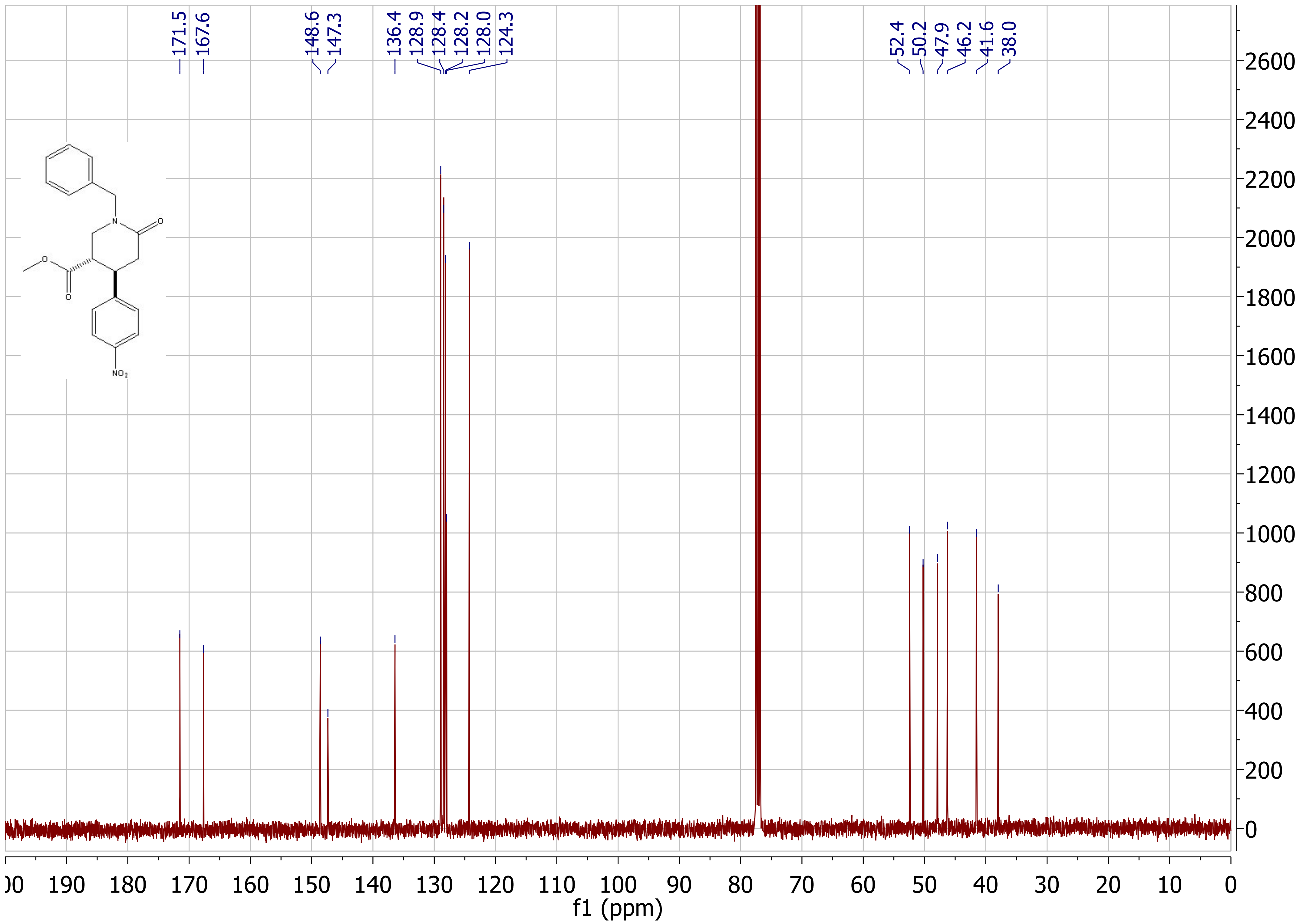

171.5
167.6
148.6
147.3
136.4
128.9
128.4
128.2
128.0
124.3
52.4
50.2
47.9
46.2
41.6
38.0
N
O
O
O
NO2
f1 (ppm)

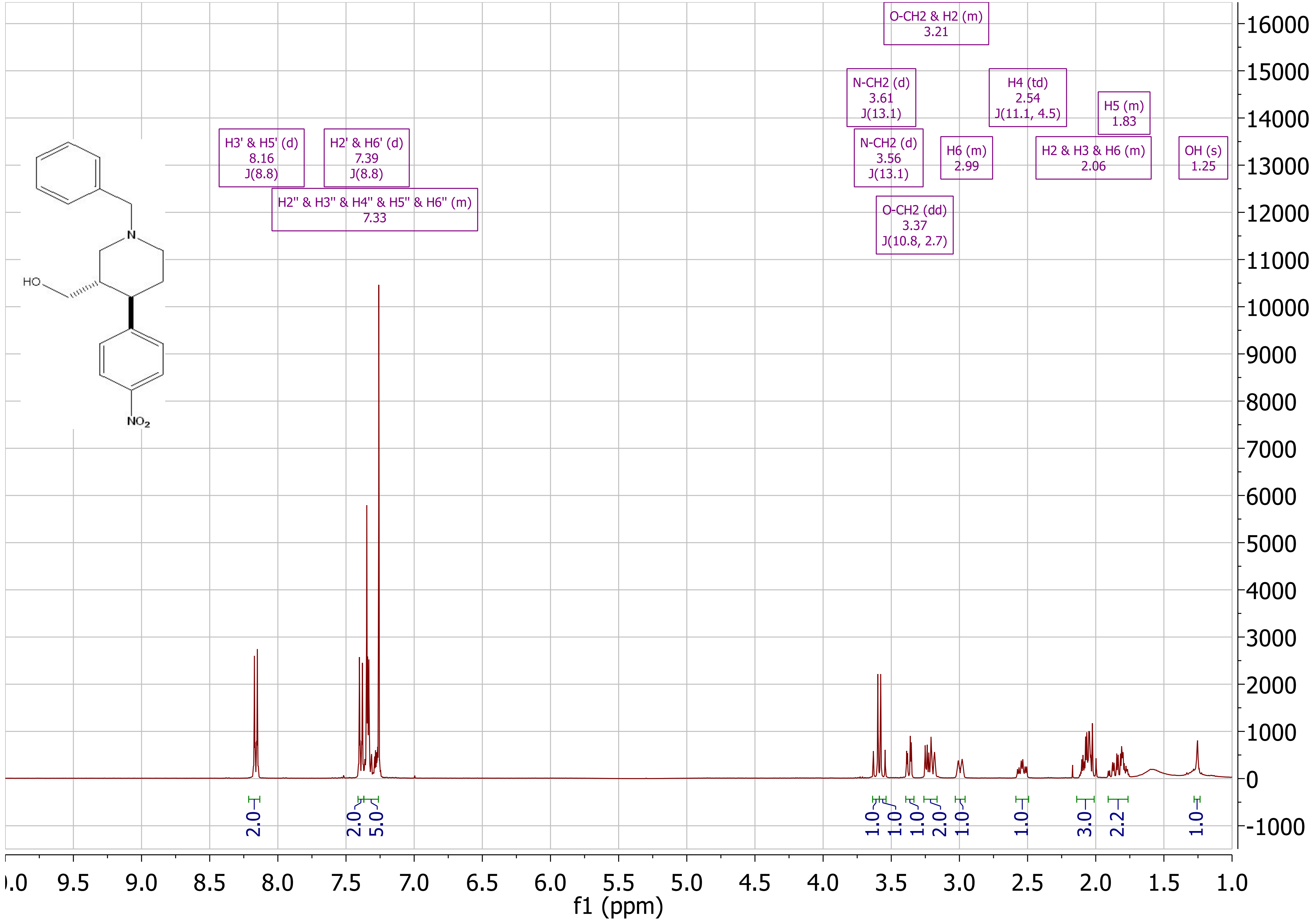

HO
N
NO2
H3' & H5' (d)
8.16
J(8.8)
H2' & H6' (d)
7.39
J(8.8)
H2'' & H3'' & H4'' & H5'' & H6'' (m)
7.33
O-CH2 & H2 (m)
3.21
N-CH2 (d)
3.61
J(13.1)
N-CH2 (d)
3.56
J(13.1)
O-CH2 (dd)
3.37
J(10.8, 2.7)
H6 (m)
2.99
H4 (td)
2.54
J(11.1, 4.5)
H5 (m)
1.83
H2 & H3 & H6 (m)
2.06
OH (s)
1.25
2.0
2.0
5.0
1.0
1.0
1.0
2.0
1.0
1.0
3.0
2.2
1.0
16000
15000
14000
13000
12000
11000
10000
9000
8000
7000
6000
5000
4000
3000
2000
1000
0
-1000
9.5
9.0
8.5
8.0
7.5
7.0
6.5
6.0
5.5
5.0
4.5
4.0
3.5
3.0
2.5
2.0
1.5
1.0
f1 (ppm)

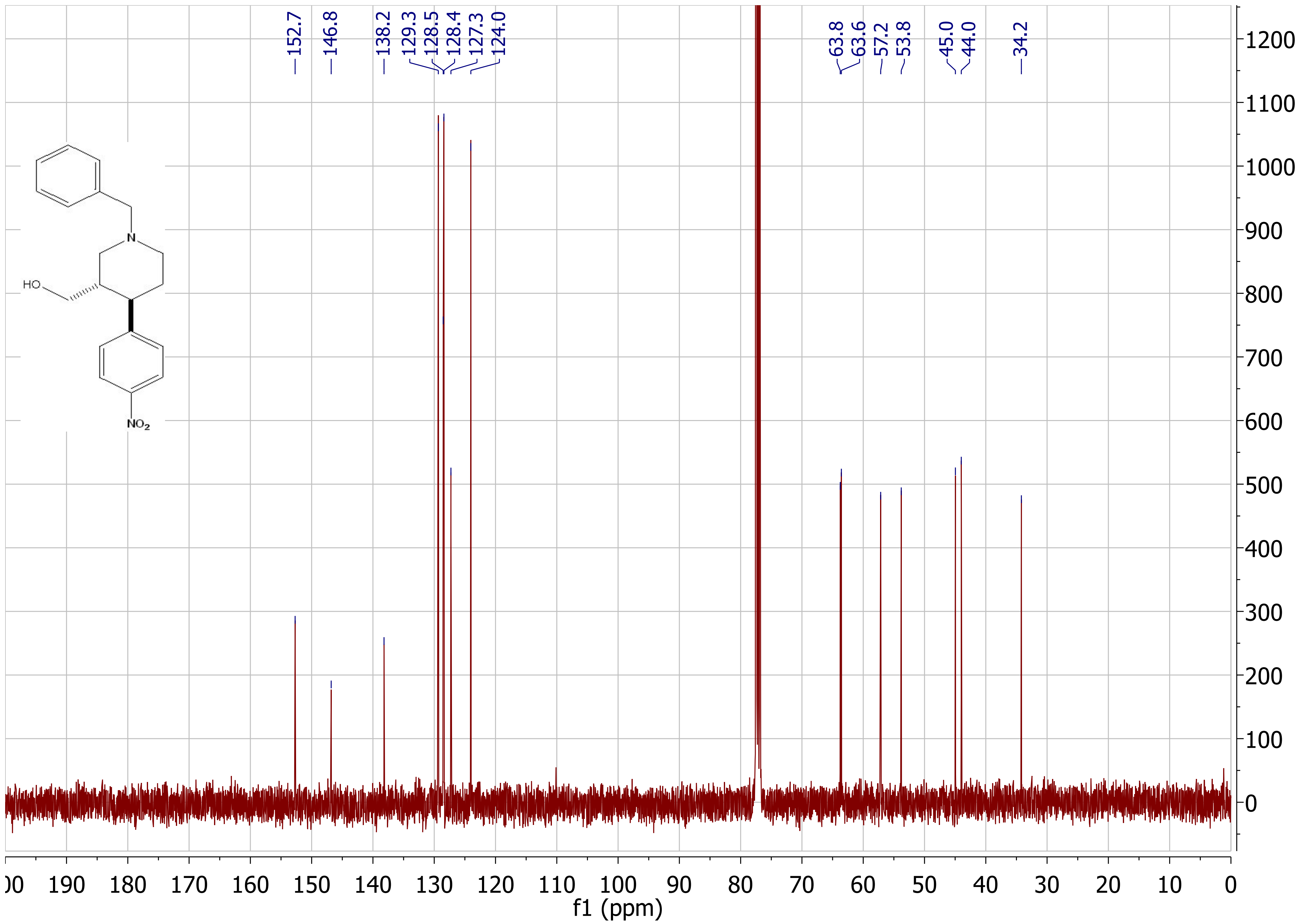

152.7
146.8
138.2
129.3
128.5
128.4
127.3
124.0
63.8
63.6
57.2
53.8
45.0
44.0
34.2
HO
N
NO2
1200
1100
1000
900
800
700
600
500
400
300
200
100
0
190
180
170
160
150
140
130
120
110
100
90
80
70
60
50
40
30
20
10
0
f1 (ppm)

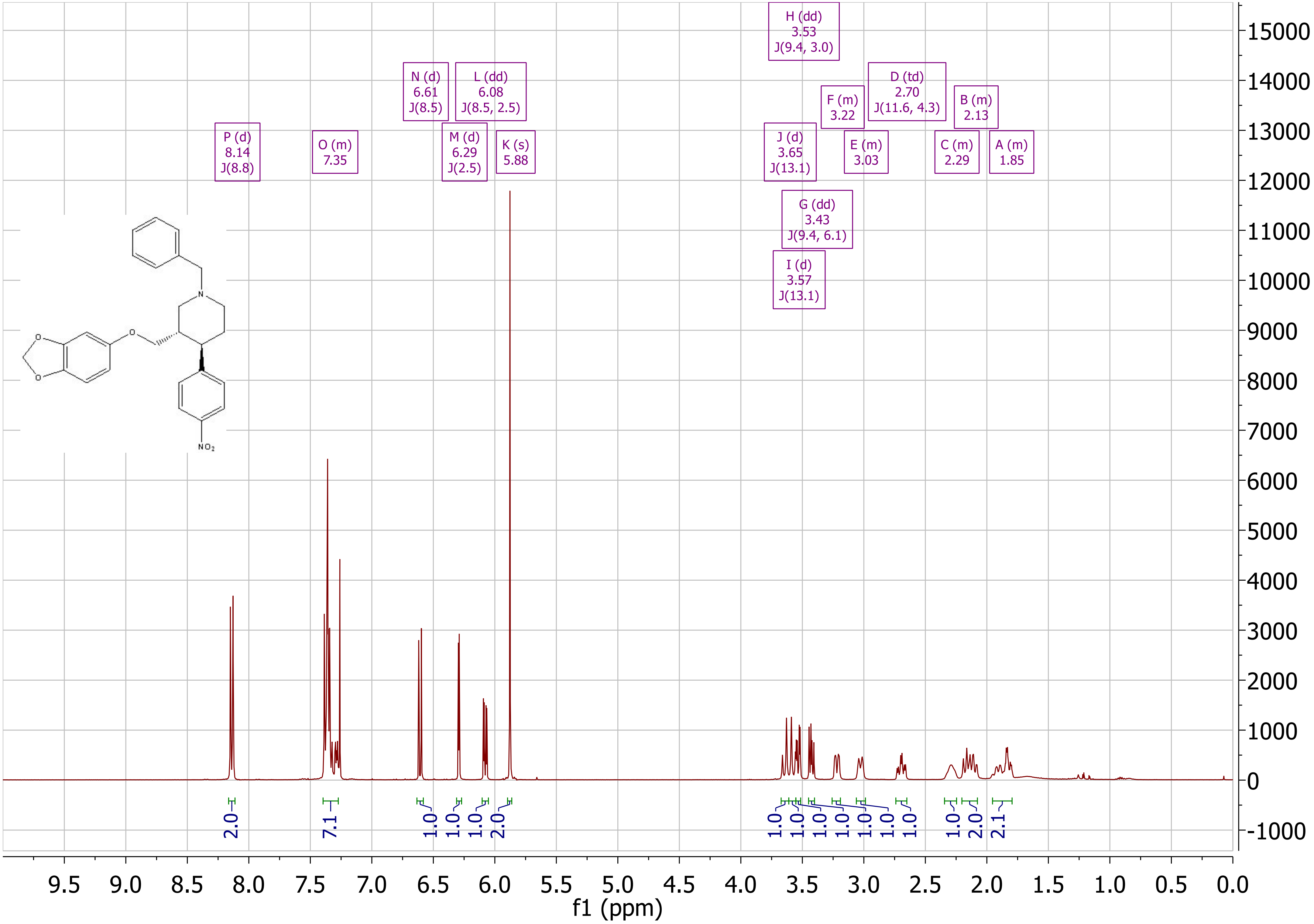

P (d) 8.14 J(8.8)
O (m) 7.35
N (d) 6.61 J(8.5)
M (d) 6.29 J(2.5)
L (dd) 6.08 J(8.5, 2.5)
K (s) 5.88
J (d) 3.65 J(13.1)
I (d) 3.57 J(13.1)
H (dd) 3.53 J(9.4, 3.0)
G (dd) 3.43 J(9.4, 6.1)
F (m) 3.22
E (m) 3.03
D (td) 2.70 J(11.6, 4.3)
C (m) 2.29
B (m) 2.13
A (m) 1.85
NO_2
2.0 7.1 1.0 1.0 1.0 2.0 1.0 1.0 1.0 1.0 1.0 1.0 1.0 1.0 2.0 2.1
f1 (ppm)

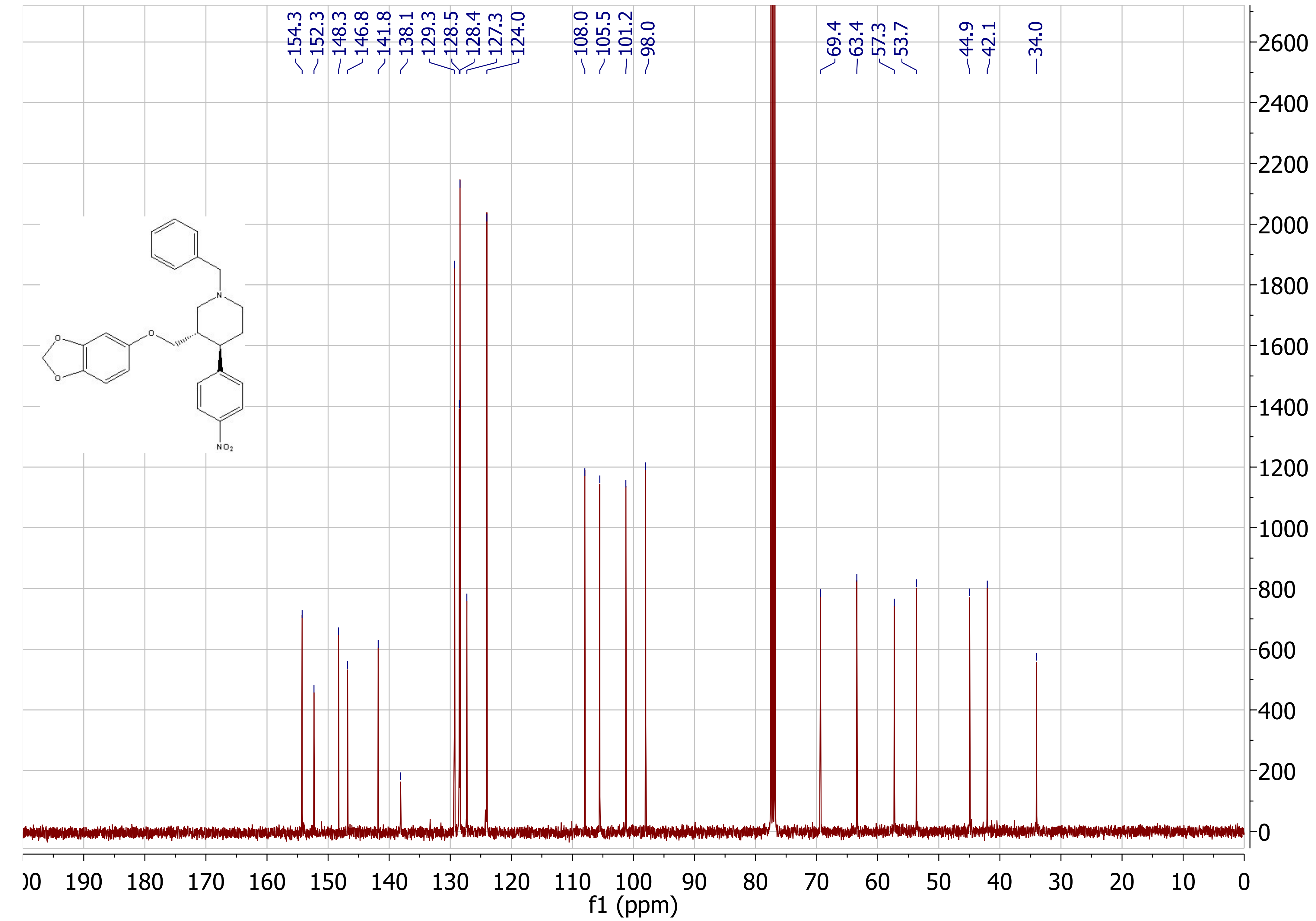

154.3
152.3
148.3
146.8
141.8
138.1
129.3
128.5
128.4
127.3
124.0
108.0
105.5
101.2
98.0
69.4
63.4
57.3
53.7
44.9
42.1
34.0
N
O
O
O
NO2
2600
2400
2200
2000
1800
1600
1400
1200
1000
800
600
400
200
0
190
180
170
160
150
140
130
120
110
100
90
80
70
60
50
40
30
20
10
0
f1 (ppm)

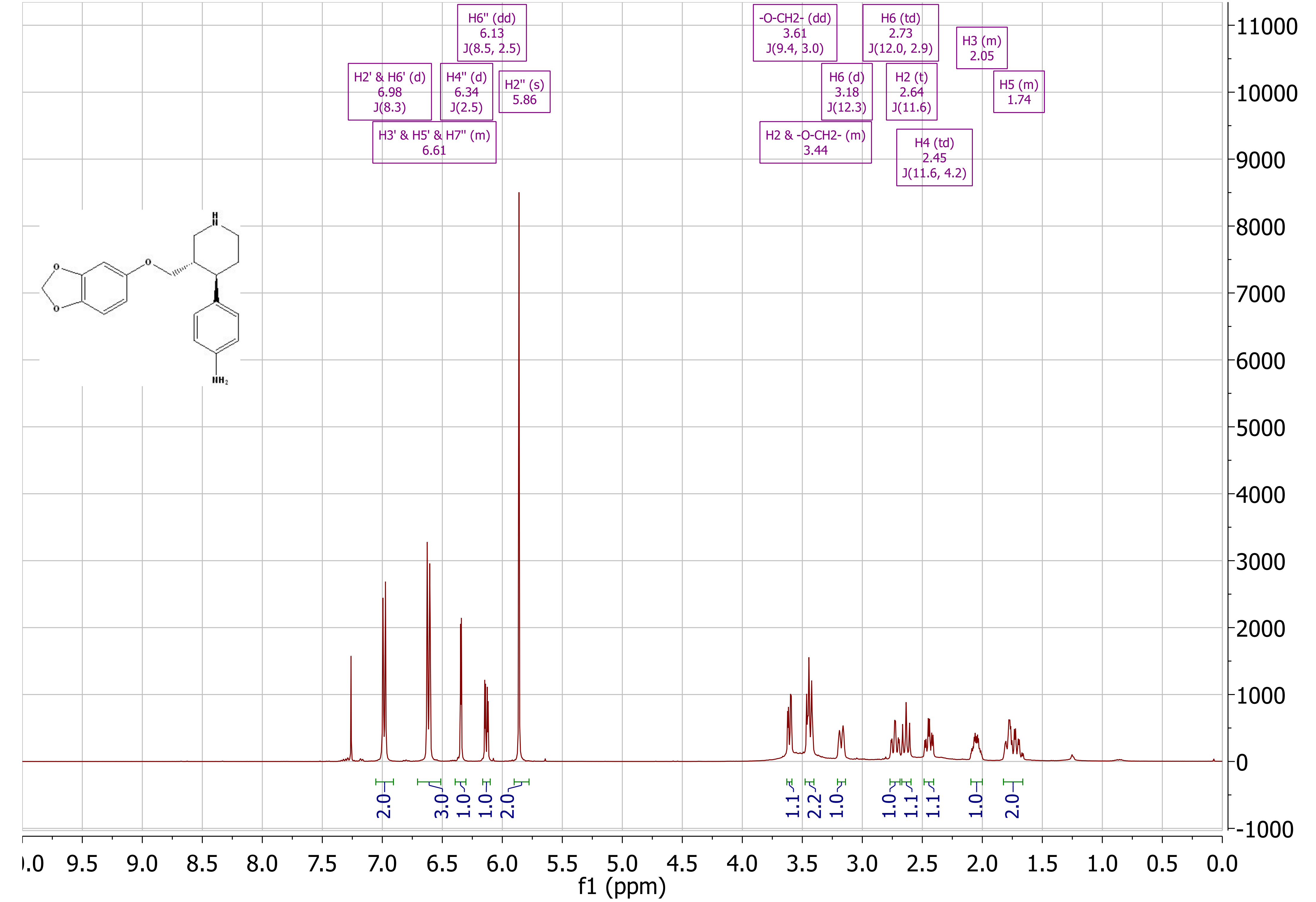

H2' & H6' (d)
6.98
J(8.3)
H3' & H5' & H7'' (m)
6.61
H4'' (d)
6.34
J(2.5)
H6'' (dd)
6.13
J(8.5, 2.5)
H2'' (s)
5.86
-O-CH2- (dd)
3.61
J(9.4, 3.0)
H2 & -O-CH2- (m)
3.44
H6 (d)
3.18
J(12.3)
H6 (td)
2.73
J(12.0, 2.9)
H2 (t)
2.64
J(11.6)
H4 (td)
2.45
J(11.6, 4.2)
H3 (m)
2.05
H5 (m)
1.74
2.0
3.0
1.0
1.0
2.0
1.1
2.2
1.0
1.0
1.1
1.1
1.0
2.0
f1 (ppm)

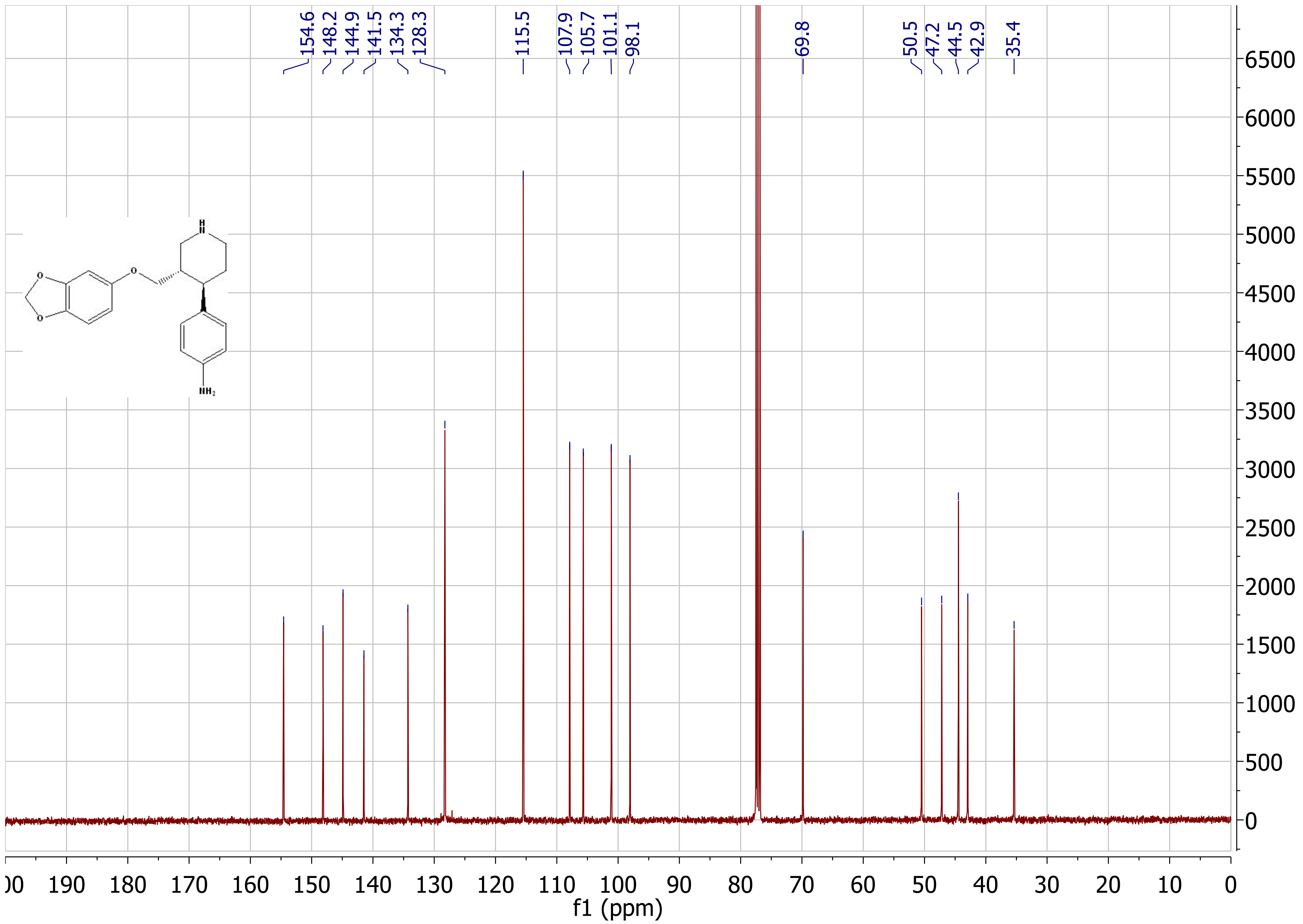

154.6
148.2
144.9
141.5
134.3
128.3
115.5
107.9
105.7
101.1
98.1
69.8
50.5
47.2
44.5
42.9
35.4
NH2
f1 (ppm)

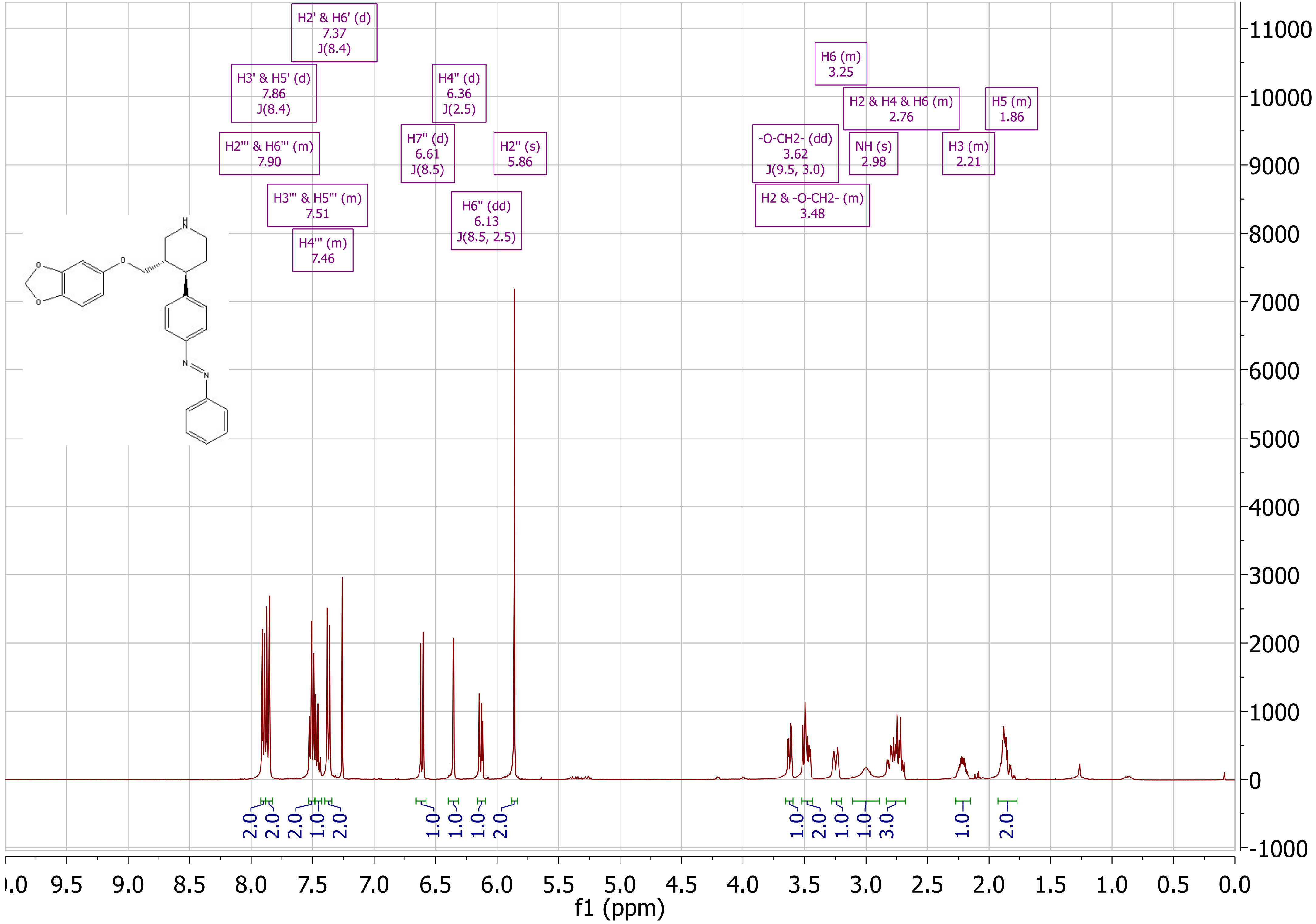

H2' & H6' (d)
7.37
J(8.4)
H3' & H5' (d)
7.86
J(8.4)
H2''' & H6''' (m)
7.90
H3''' & H5''' (m)
7.51
H4''' (m)
7.46
H4'' (d)
6.36
J(2.5)
H7'' (d)
6.61
J(8.5)
H2'' (s)
5.86
H6'' (dd)
6.13
J(8.5, 2.5)
H6 (m)
3.25
H2 & H4 & H6 (m)
2.76
H5 (m)
1.86
-O-CH2- (dd)
3.62
J(9.5, 3.0)
NH (s)
2.98
H3 (m)
2.21
H2 & -O-CH2- (m)
3.48
2.0
2.0
2.0
1.0
2.0
1.0
1.0
1.0
2.0
1.0
2.0
1.0
1.0
3.0
1.0
2.0
f1 (ppm)

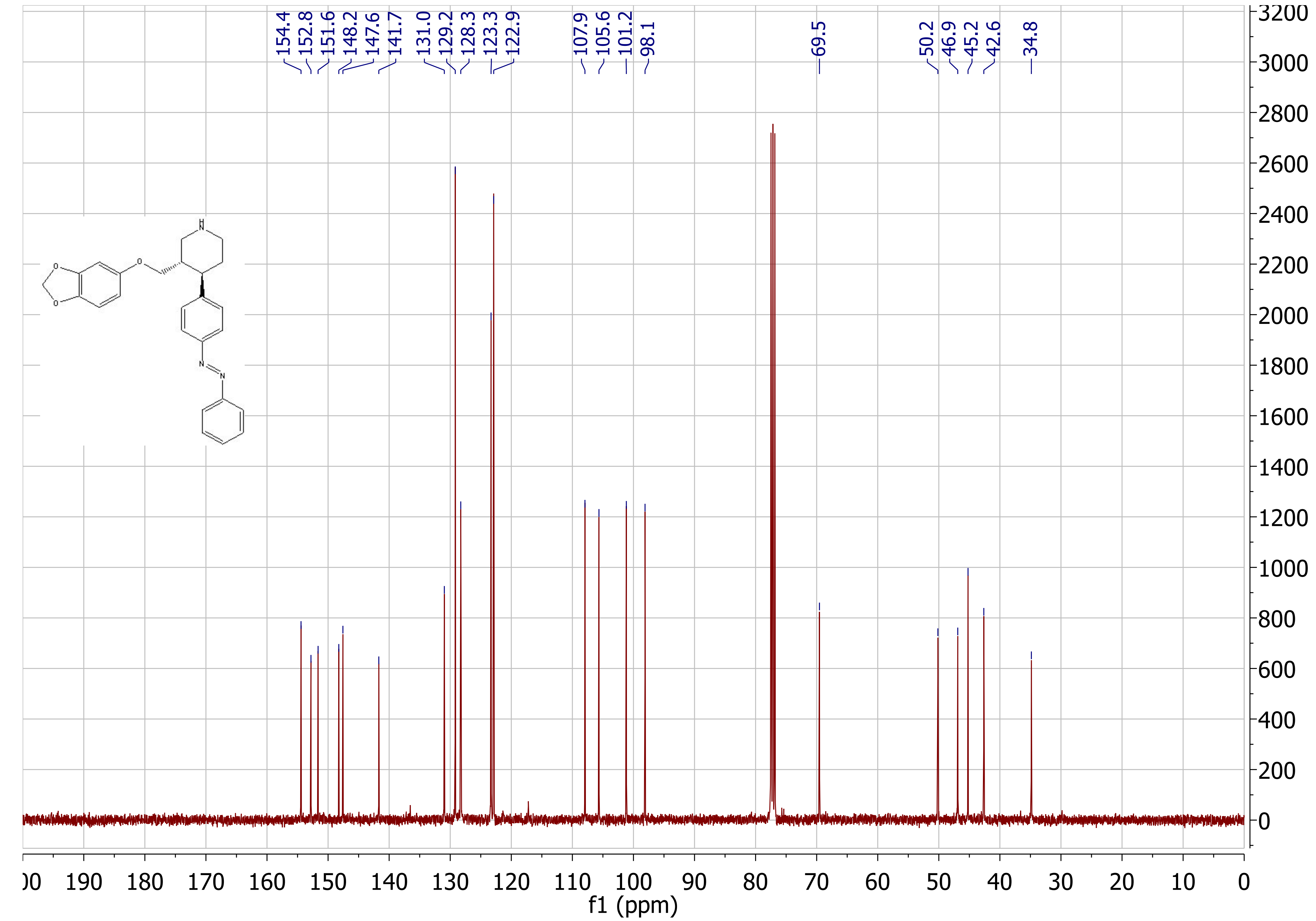

154.4
152.8
151.6
148.2
147.6
141.7
131.0
129.2
128.3
123.3
122.9
107.9
105.6
101.2
98.1
69.5
50.2
46.9
45.2
42.6
34.8
3200
3000
2800
2600
2400
2200
2000
1800
1600
1400
1200
1000
800
600
400
200
0
190
180
170
160
150
140
130
120
110
100
90
80
70
60
50
40
30
20
10
0
f1 (ppm)
